# Type-Specific HPV Prevalence in Cervical Cancer and High-Grade Lesions in Latin America and the Caribbean: Systematic Review and Meta-Analysis

**DOI:** 10.1371/journal.pone.0025493

**Published:** 2011-10-04

**Authors:** Agustín Ciapponi, Ariel Bardach, Demián Glujovsky, Luz Gibbons, María Alejandra Picconi

**Affiliations:** 1 Institute for Clinical Effectiveness and Health Policy (IECS), Buenos Aires, Argentina; 2 Oncogenic Viruses Service, National Reference Laboratory of HPV, National Institute of Infectious Diseases, ANLIS, Buenos Aires, Argentina; Health Protection Agency, United Kingdom

## Abstract

**Background:**

Cervical cancer is a major public health problem in Latin America and the Caribbean (LA&C), showing some of the highest incidence and mortality rates worldwide. Information on HPV type distribution in high-grade cervical lesions (HSIL) and invasive cervical cancer (ICC) is crucial to predict the future impact of HPV16/18 vaccines and screening programmes, and to establish an appropriate post-vaccinal virologic surveillance. The aim was to assess the prevalence of HPV types in HSIL and ICC in studies in LA&C.

**Methods and Findings:**

We performed a systematic review, following the MOOSE guidelines for systematic reviews of observational studies, and the PRISMA statement for reporting systematic reviews and meta-analyses. Inclusion criteria were at least ten cases of HSIL/ICC, and HPV-type elicitation. The search, without language restrictions, was performed in MEDLINE, Cochrane Library, EMBASE, LILACS from inception date to December 2009, proceedings, reference lists and consulting experts. A meta-analysis was performed using arc-sine transformations to stabilize the variance of simple proportions. Seventy-nine studies from 18 countries were identified, including 2446 cases of HSIL and 5540 of ICC. Overall, 46.5% of HSIL cases harbored HPV 16 and 8.9% HPV18; in ICC, 53.2% of cases harbored HPV 16 and13.2% HPV 18. The next five most common types, in decreasing frequency, were HPV 31, 58, 33, 45, and 52.

Study's limitations comprise the cross-sectional design of most included studies and their inherent risk of bias, the lack of representativeness, and variations in the HPV type-specific sensitivity of different PCR protocols.

**Conclusions:**

This study is the broadest summary of HPV type distribution in HSIL and ICC in LA&C to date. These data are essential for local decision makers regarding HPV screening and vaccination policies. Continued HPV surveillance would be useful, to assess the potential for changing type-specific HPV prevalence in the post-vaccination era in Latin America.

## Introduction

Human papillomavirus infection (HPV) is one of the most common sexually transmitted diseases worldwide [1]. Infection by certain types of HPV is recognized as a causal and necessary factor in the development of cervical cancer [2]. Cervical cancer represents the second-most common malignancy in women around the world and contributes to 9.8% of all female cancers [3]. Cervical cancer accounts for 10% of all female cancers, making it the second leading cause of cancer death in women. Worldwide, there were approximately 500,000 incident cases and 275,000 deaths due to cancer of the cervix in 2002. Latin America and the Caribbean accounted for 15% and 11%, respectively, of this burden [4]. The age-standardized cervical cancer incidence rate is 30.6 per 100,000 persons in Central America, and 28.6 per 100,000 persons in South America [5].

It is now recognized that virtually all cervical cancers (both the squamous and adenocarcinoma histological types) and their precursor lesions are causally related to cervical infections through at least 14 oncogenic HPV genotypes (16, 18, 31, 33, 35, 39, 45, 51, 52, 56, 58, 59, 66 and 68) [6], [7]. However, only a minority of pre-neoplastic lesions progress to cancer; the HPV type is a robust risk factor for differential progression [8]. Since cervical cancer affects relatively young women, it represents the single biggest cause of years of life lost (YLL) from cancer in the developing world, contributing more to this burden of disease measure than do tuberculosis, maternal conditions or acquired immunodeficiency syndrome (AIDS) [9]. In developed countries, Papanicolaou (PAP) smear test screening has decreased the incidence of cervical cancer by about 70% in recent decades; however, it still represents a major public health issue in LA&C because of the failure of prevention programs [4]. Previous meta-analyses have reported information about prevalence distribution of high-risk HPV types in HSIL or cervical cancer worldwide; however, this data is variable and incomplete for LA&C populations [10]–[14]. Regional data on type-distribution is essential for estimating the impact of vaccines on cervical cancer and for the development of screening programs. The aim of the present study is to assess exhaustively the HPV type distribution in HSIL and ICC in studies in LA&C region.

## Materials and Methods

We performed a systematic review, following the Meta-analysis Of Observational Studies in Epidemiology (MOOSE) guidelines [15] for systematic reviews of observational studies, and the Preferred Reporting Items for Systematic reviews and Meta-Analyses (PRISMA) statement for reporting systematic reviews and meta-analyses [16], [17], which replaced the Quality Of Reporting Of Meta-analysis (QUOROM) statement [18].

### Search methodology

A search, without language restrictions, was performed on the main international and regional literature databases MEDLINE; EMBASE; CINAHL; NHS R&D Health Technology Assessment Program; ClinicalTrials.gov; LILACS; Cab International Global Health; Pascal Biomed; generic and academic internet search and meta-search engines; and the specialized register of the Cochrane Gynecological Cancer Group from its inception date to December 2009. Databases containing regional proceedings or congress's annals, doctoral theses and experts were also consulted.

The Medline, LILACS, and EMBASE search strategy is available at the **Appendix S1**. An exhaustive strategy module was developed to localize studies from LA&C. According to pre-specified criteria, pairs of authors independently examined the title, abstract, and descriptors of the articles in order to identify potentially relevant studies for full review. The reference lists of the articles finally included were hand-searched for additional information. If data or data subsets of the same population were published in more than one article, only the publication with the largest sample size was selected, after consulting the principal investigator. Discrepancies were resolved by consensus or, finally, by a third author. The full texts of relevant articles retrieved were examined using a pre-designed form.

### Types of studies and participants

Any descriptive epidemiological study with individual-level data was considered. Participant subjects were women from LA&C countries, in studies of cervical cancer/HSIL associated with HPV. The inclusion criteria were a) to inform at least ten cases of HSIL or ICC, b) confirmed by biopsy, and c) HPV-type elicitation. We excluded those papers that undoubtedly failed to meet the aforementioned inclusion criteria. Studies using both polymerase chain reaction (PCR)-amplified and non-amplified genotyping methods were included. There were no restrictions on PCR primers' utilization. HPV DNA tissue sources included fixed or fresh biopsies and/or exfoliated cells. Outcome measures included global and type-specific HPV prevalence. Two attempts of email contact with the author were made in order to recover missing data.

### Methodological quality assessment

Two reviewers assessed the methodological quality of studies independently. Discrepancies were solved by consensus of the whole team. Observational studies or control arms of randomized controlled trials were assessed by a checklist of essential items stated in STROBE [19] (Strengthening the Reporting of Observational studies in Epidemiology) statement, two methodological papers [20], [21] and the general guidelines of MOOSE [15]. (See **Appendix S2**)

Pairs of reviewers independently abstracted the following key information: country where the samples were drawn, setting, population, sample size, study design, age, study year, distribution of cases by histological type, type of cervical specimen and PCR primers, type-specific and overall prevalence of HPV infection, reported duration of HPV infection, and quality score. Data on HPV-specific prevalence was extracted independently for squamous cell carcinoma (SCC) and for adeno- and adenosquamous carcinoma. Each study, or regional components of a study, was classified by the following criteria: 1) geographical region (Central America/Mexico/The Caribbean or South America) 2) income level as defined by the Gross Nation Income (GNI) World Bank Classification (lower-middle income, upper-middle income, high income), 3) tissue source (exfoliated cells, fixed biopsies, fresh biopsies, combined), and 4) genotyping method (Southern blot, Dot blot, FISH and In Situ Hybridization), PCR 1 (PCR MY09/11 or Consensus primers), PCR 2 (PCR SPF, GP5/6, E6, E7 and others) and PCR 3 (PCR MY and GP performed together).

### Statistical analysis

HPV prevalence data was expressed as a percentage of all cases tested for HPV. Multiple infections were separated into constituent types, thus type-specific prevalence represents both single and multiple infections. For HPV type-specific prevalence, only studies testing for a particular HPV type contribute to the analysis for that type, and therefore sample size varied between the type-specific analyses. In order to perform a meta-analysis with prevalence data, we first transformed proportions into a quantity (the Freeman-Tukey variant of the arcsine square root transformed proportion) [22]. The pooled proportion was calculated as the back-transformation of the weighted mean of the transformed proportions, using inverse arcsine variance weights for the fixed effects model. The arcsine transformations were necessary to stabilize the variance of simple proportions.

One must consider that each HPV type proportion is a pooled estimate of only those studies reporting the particular HPV type. Hence, each proportion has its own denominator and must be considered regardless of the other types. Thus, cumulative point estimates do not sum to 100%. DerSimonian-Laird weights for the random effects model [23] were applied where heterogeneity between studies was found. The I^2^ statistic quantifies the heterogeneity between studies. This statistic describes the percentage of the variability in effect estimates that is due to heterogeneity rather than sampling error (chance). [24] We used Statsdirect and STATA 8.0.

We hypothesized the following possible sources of heterogeneity: age, risk factors of HPV and/or HSIL/cervical cancer, country, geographical region, income level by the Gross National Income (GNI) World Bank Classification, type of cervical lesion, type of tissue source and type of genotyping method used. With the available data we could perform pre-designed subgroup analyses considering the country where the study was carried out, the geographical region, the income level of the country according the Gross National Income (GNI) World Bank Classification, the type of genotyping method and the tissue source. Additionally, we applied a meta-regression analysis in order to further study the possible sources of heterogeneity and to get the adjusted prevalence. Publication bias was unlikely as assessed by funnel plots although this type of bias is unlikely to occur in prevalence studies (data not shown). No ethical approval was required for this study.

## Results

The present Systematic Review and Meta-analysis met the PRISMA statement requirements (See **Checklist S1** and **Diagram S1**).

**Figure 1:**
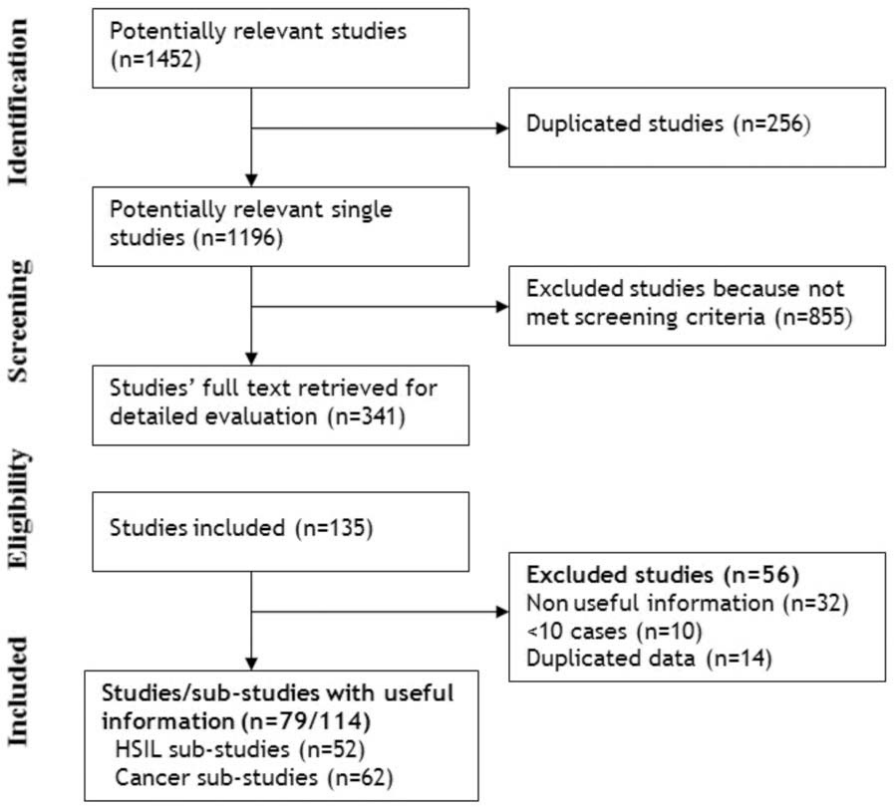
Study flow diagram.

Overall, 1452 citations were retrieved from the search strategy. After the assessment (**Figure 1**), 79 studies from 18 countries, totaling 7986 women, met the inclusion criteria [25]–[100]. Study characteristics are presented in **Appendix S3**. Nine countries were from Central America/Mexico/The Caribbean (31.8% of the women) and nine countries from South America (68.2%). One country, was a high-income nation (0.3% of women), six countries were middle-income (72.3%), and eleven countries were low-income (27.4%).

We considered 114 sub-studies for the analysis, including seven country-level sub-studies from Bosch 1995 [29] and discriminated sub-studies by cervical lesion (52 sub-studies evaluated patients with HSIL and 62 evaluated patients with cervical cancer). Thirteen studies had a moderate risk of bias [29], [41], [44], [68], [70], [73], [82], [92], [101]–[105] and the rest carried a high risk of bias. HPV DNA was retrieved from fixed biopsies in 34.2%, from exfoliated cells in 34.2%, from fresh biopsies in 19.7%, and from exfoliated cells and fresh biopsies in 11.8% of the studies. Most of the authors used PCR MY09/11 or non-specified consensus primers (n = 30), while the rest used membrane or in-situ hybridization (n = 9), PCR GP5/6 or SPF or others (i.e. E6 and E7) (n = 30), or PCR using MY and GP together (n = 8) (**Appendix S3**).

HSIL/ICC cases came mainly from Brazil (23.7%), Argentina (19.0%), and Mexico (17.9%). The HSIL and ICC prevalence, and ICC∶HSIL prevalence ratio by type are presented in **Table 1**. HPV16 and HPV18, were the first- and second-most common types, respectively for both HSIL and ICC. HPV18, 45 and 16 had the highest ICC∶HSIL prevalence ratio (1.48, 1.18, and 1.14 respectively). Conversely, HPV11, 56, 6, 68 and 58, were each 2 to 3-fold more prevalent in HSIL than in ICC.

**Table 1:** HSIL and CANCER prevalence by HPV type.

HPV TYPE	HSIL		CANCER		CANCER∶HSIL
	N° of patients	Prevalence %	N° of patients	Prevalence %	Prevalence
	(N° of Studies)	(95% CI)	(N° of Studies)	(95% CI)	ratio
**Global**	**2446 (52)**		**5540 (62)**		
**Any**	1749 (36)	82.5 (77.3–87.1)	3435 (43)	89.0 (84.3–92.9)	1.08
**Type 6**	1415 (29)	4.2 (2.2–6.7)	2274 (32)	1.7 (0.9–2.8)	0.4
**Type 11**	1414 (29)	2.4 (1.3–3.8)	2274 (32)	1.3 (0.5–2.5)	0.54
**Type 16**	2327 (49)	46.5 (41.3–51.7)	5463 (60)	53.2 (49.1–57.2)	1.14
**Type 18**	2194 (45)	8.9 (6.3–11.8)	4962 (56)	13.2 (11.0–15.6)	1.48
**Type 31**	1785 (36)	8.0 (6.0–10.4)	3903 (45)	7.5 (5.5–9.8)	0.94
**Type 33**	1722 (35)	6.5 (4.7–8.5)	3821 (42)	4.3 (3.2–5.5)	0.66
**Type 35**	1228 (24)	3.0 (1.9–4.4)	2332 (31)	2.0 (1.3–2.7)	0.67
**Type 39**	885 (20)	2.4 (1.5–3.5)	1977 (27)	1.8 (1.3–2.4)	0.75
**Type 45**	1077 (24)	3.9 (2.8–5.2)	3389 (37)	4.6 (3.5–5.7)	1.18
**Type 51**	1013 (21)	3.7 (2.1–5.7)	2131 (30)	2.1 (1.1–3.3)	0.57
**Type 52**	1152 (25)	4.9 (2.9–7.4)	2544 (34)	3.2 (2.1–4.4)	0.65
**Type 56**	892 (19)	2.4 (1.5–3.4)	2155 (28)	1.2 (0.8–1.7)	0.5
**Type 58**	1197 (26)	8.7 (6.0–11.9)	2564 (34)	3.0 (2.1–4.1)	0.34
**Type 59**	954 (21)	1.9 (1.2–2.9)	2199 (30)	1.6 (1.1–2.2)	0.84
**Type 66**	926 (20)	1.8 (1.1–2.8)	2095 (28)	1.1 (0.7–1.6)	0.61
**Type 68**	619 (14)	1.3 (0.6–2.3)	1864 (23)	0.5 (0.3–0.9)	0.38
Other*	1479 (32)	11.6 (7.6–16.2)	3177 (34)	7.5 (5.0–10.4)	0.65
**Multiple**	1431 (29)	16.8 (12.9–21.2)	2090 (27)	12.6 (8.7–17.2)	0.75

**Figure 2:**
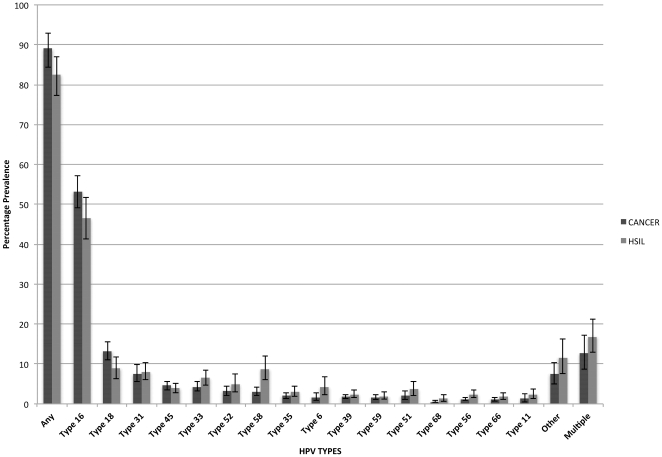
HPV type-specific prevalence in Cancer and HSIL, with 95% CIs.

The comparison of HPV type-specific prevalence cancer and HSIL cases is illustrated by **Figure 2**.

### High grade intraepithelial lesions (HSIL)

In the 52 sub-studies included in the HSIL systematic review, 16 were performed in Mexico or Central America and 36 in South America. Overall, a total of 2446 patients' samples were analyzed with a median of 47.5 specimens in each sub-study (range 6 to 130). Most data came from cross-sectional studies (n = 39) while seven came from case-control studies, four from cohort studies/prospective follow up, one from a nested case-control study, one from a before-after study and one from a randomized controlled trial. Mean age of women was 40.4±7.6 years old.

**Figure 3:**
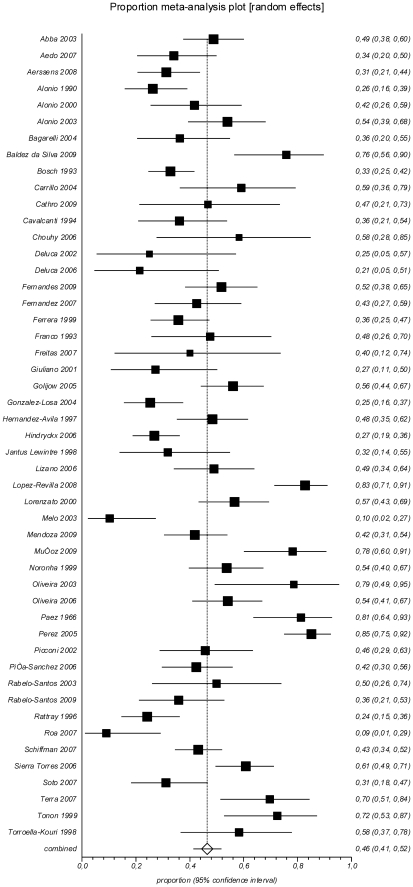
Prevalence of HPV16 in HSIL.

Any HPV in HSIL was found in a pooled proportion of 82.5% (95% CI 77.3–87.1%; I^2^ = 86.4%) of samples, while prevalence of HPV16 was 46.5% (95% CI 41.3–51.7%; I^2^ = 84.6%) and prevalence of HPV18 was 8.9% (95% CI 6.3–11.8%; I^2^ = 80.0%) (**Table 1** **,** **Figure 3** **,** **4**). Multiple HPV infections were seen in 16.8% (95% CI 12.8–21.4; I^2^ = 77.0%) of the analyzed samples.

**Figure 4:**
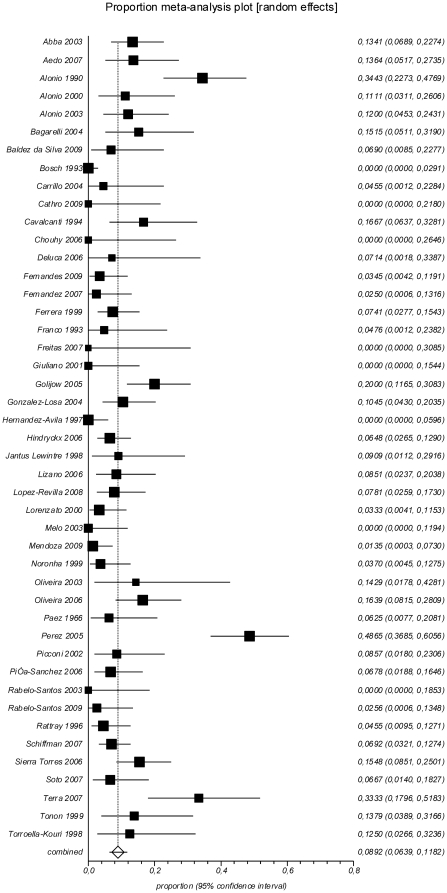
Prevalence of HPV18 in HSIL.

**Table 2** presents the HPV16/18 prevalence in ICC and HSIL by country, region, and Gross National Income (GNI) from the World Bank's classification. In Argentina (12 studies) the pooled prevalence of HPV16 in HSIL samples was 48.5% (95% CI 36.7–60.3%; I^2^ = 85.8%). In Brazil (13 studies) the pooled prevalence of HPV16 in HSIL samples was 52.7% (95% CI 45.6–59.6%; I^2^ 56.8%). In Mexico (9 studies), the pooled prevalence of HPV16 in HSIL samples was 48.5% (95% CI 35.5–61.6%; I^2^ 86.1%).

**Table 2:** HPV16/18 prevalence in ICC and HSIL: subgroup analysis by country, region, and GNI World Bank classification.

Subgroups	HSIL				CERVICAL CANCER			
	HPV TYPE: 16		HPV TYPE: 18		HPV TYPE: 16		HPV TYPE: 18	
	N patients(studies)	Prevalence(95% CI)	N patients(studies)	Prevalence(95% CI)	N patients(studies)	Prevalence(95% CI)	N patients(studies)	Prevalence(95% CI)
**GLOBAL**	**2327** (49)	**46.5** (41.3–51.7)	**2194** (45)	**8.9** (6.3–11.8)	**5463** (60)	**53.2** (49.1–57.2)	**4962** (56)	**13.2** (11–15.6)
**By country**								
Argentina	502 (12)	48.5 (36.7–60.3)	490 (11)	16.9 (9.8–25.4)	1013 (10)	59.5 (51.3–67.5)	1013 (10)	17.6 (12–24.1)
Barbados	-	-	-	-	21 (1)	71.4 (47.8–88.7)	-	-
Belize	15 (1)	46.7 (21.3–73.4)	15 (1)	0 (0–0)	-	-	-	-
Bolivia	-	-	-	-	49 (1)	34.7 (21.7–49.6)	49 (1)	4.1 (0.5–14)
Brazil	466 (13)	52.7 (45.6–59.6)	466 (13)	9 (5–14.1)	1269 (13)	53.2 (42.9–63.3)	1269 (13)	15.8 (8.9–24.2)
Chile	95 (3)	18.5 (5.8–36.3)	73 (2)	5.9 (0.2–26.2)	420 (4)	51.8 (29.7–73.5)	420 (4)	9.5 (4.2–16.7)
Colombia	241 (3)	56.7 (31.2–80.4)	209 (2)	4.9 (1.7–29.5)	450 (4)	46.7 (35.9–57.7)	450 (4)	7.5 (3.7–12.6)
Costa Rica	130 (1)	43.1 (34.4–52)	130 (1)	7.4 (2.8–15.4)	35 (1)	45.7 (28.8–63.4)	35 (1)	17.1 (6.6–33.6)
Cuba	45 (1)	31.1 (18.2–46.6)	45 (1)	6.3 (0.8–20.8)	45 (1)	57.8 (42.2–72.3)	45 (1)	6.7 (1.4–18.3)
Ecuador	32 (1)	81.3 (63.6–92.8)	32 (1)	4.5 (0.9–12.7)	47 (1)	80.9 (66.7–90.9)	47 (1)	4.3 (0.5–14.5)
Honduras	81 (1)	35.8 (25.4–47.2)	81 (1)	6.9 (3.2–12.7)	104 (1)	43.3 (33.6–53.3)	104 (1)	10.6 (5.4–18.1)
Jamaica	66 (1)	24.2 (14.5–36.4)	66 (1)	6.7 (1.4–18.3)	-	-	-	-
Mexico	405 (9)	48.5 (35.5–61.6)	405 (9)	6 (3.1–9.7)	1021 (14)	54.9 (47.6–61.9)	840 (13)	12.8 (9.7–16.2)
Nicaragua	175 (2)	28.8 (22.4–35.7)	108 (1)	6.7 (1.4–18.3)	136 (2)	38.1 (17–61.9)	19 (1)	5.3 (0.1–26)
Panama	-	-	-	-	255 (2)	41.6 (31.3–52.2)	73 (1)	15.1 (7.8–25.4)
Paraguay	74 (1)	41.9 (30.5–53.9)	74 (1)	1.4 (0–7.3)	154 (2)	61.3 (33.9–85.2)	154 (2)	7.2 (1.8–15.7)
Peru	-	-	-	-	198 (1)	55.6 (48.3–62.6)	198 (1)	12.6 (8.3–18.1)
Suriname	-	-	-	-	246 (2)	42.2 (29.4–55.7)	246 (2)	16.3 (12–21.2)
**By geographic region**								
Central America and Mexico	917 (16)	41.7 (33.8–49.8)	850 (15)	6.3 (4.6–8.3)	1617 (22)	51.7 (45.6–57.8)	1116 (18)	12.5 (10.1–15.1)
South America	1410 (33)	48.9 (42.2–55.5)	1344 (30)	10.5 (6.6–15.1)	3846 (38)	54.0 (48.6–59.2)	3846 (38)	13.3 (10.4–16.5)
**By GNI World Bank classification**								
Lower middle income	714 (10)	43.6 (32.8–54.8)	615 (8)	5.5 (2.3–10.1)	1429 (15)	49.4 (42.6–56.2)	1312 (14)	9.5 (7.2–12)
Upper middle income	1613 (39)	47.3 (41.5–53.2)	1579 (37)	9.8 (6.8–13.2)	4013 (44)	54.1 (49.2–58.9)	3650 (42)	14.8 (11.9–18)
High income	-	-	-	-	21 (1)	71.4 (47.8–88.7)	-	-

We found a pooled prevalence of HPV18 in HSIL of 16.9% (95% CI 9.8–25.4%; I^2^ 81.2%) in Argentina, 9.0% (95% CI 5.0–14.1%; I^2^ = 66.0%) in Brazil, and 6% (95% CI 3.1–9.7%; I^2^ = 50.6%) in Mexico. HPV prevalence according to subgroups of geographic region and by GNI World Bank Classification are shown in **Table 2**. The subgroup analyses by primers used and by tissue source are shown in **Table 3**.

**Table 3:** HPV16/18 prevalence in ICC and HSIL: subgroup analysis by genotyping method and tissue source.

Subgroups	HSIL				CERVICAL CANCER			
	HPV TYPE: 16		HPV TYPE: 18		HPV TYPE: 16		HPV TYPE: 18	
	N patients(studies)	Prevalence(95% CI)	N patients(studies)	Prevalence(95% CI)	N patients(studies)	Prevalence(95% CI)	N patients(studies)	Prevalence(95% CI)
**By Genotyping Method**								
Hybridization techniques*	494 (8)	37.1 (31.6–42.7)	427 (7)	8.2 (1.6–19.3)	998 (15)	47.7 (39.1–56.4)	816 (14)	12.0 (9–15.4)
PCR 1**	948 (23)	48.2 (39.7–56.7)	882 (20)	7.6 (6–9.4)	1355 (18)	58.5 (51.2–65.7)	1174 (17)	11.3 (7.5–15.7)
PCR 2†	560 (12)	42.9 (33.5–52.7)	560 (12)	7.5 (4.2–11.6)	2618 (19)	49.9 (42.8–56.9)	2480 (17)	14.9 (10.2–20.3)
PCR 3‡	292 (5)	57.7 (39.7–74.6)	294 (5)	16.6 (4.7–33.7)	420 (6)	62.4 (51.9–72.4)	420 (6)	16.9 (11.7–22.9)
**By tissue source**								
Exfoliated cells	1330 (26)	44.7 (38.4–51.1)	1251 (24)	6.5 (4.3–9.2)	914 (16)	58.4 (52.3–64.4)	914 (16)	12.2 (8.4–16.5)
Fixed biopsies	805 (13)	43.4 (31.4–55.7)	586 (12)	13.2 (6.3–22.3)	2352 (30)	52.4 (46.2–58.6)	2149 (28)	14.6 (10.9–18.8)
Fresh biopsies	266 (7)	50.5 (36.1–64.7)	266 (7)	9.1 (4.5–15.2)	1592 (9)	50.7 (42.3–59)	1411 (8)	8.8 (6.3–11.8)
Combined	32 (1)	78.1 (60–90.7)	-	-	605 (5)	46.5 (25.6–68)	488 (4)	16.3 (10.2–23.3)

*Southern blot, Dot blot, FISH and In Situ Hybridization.

**Polymerase Chain Reaction MY09/11 or Consensus primers.

† Polymerase Chain Reaction SPF, GP5/6, E6, E7 and others.

‡ Polymerase Chain Reaction MY and GP performed together.

### Cervical cancer

In the 62 sub-studies included in the ICC systematic review, a total of 5540 patients' samples were analyzed with a median of 56 specimens in each study (range 14 to 750). Most data came from cross-sectional studies (n = 52) while 10 came from case-control studies, and one nested case-control study. Mean age of women was 41.1±7.0 years old.

**Figure 5:**
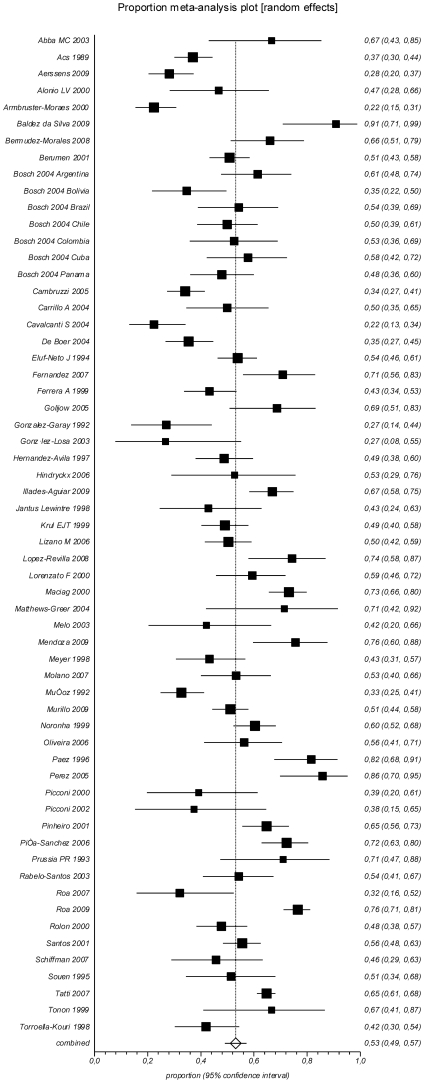
Prevalence of HPV16 in ICC.

Any HPV in cervical cancer was found in a pooled proportion of 89.0% (CI 84.3–92.9%; I^2^ = 94.0%) of the samples, while the prevalence of HPV16 was 53.2% (CI 49.1–57.2%; I^2^ = 88.5%) and the prevalence of HPV18 was 13.2% (CI 11.0–15.6%; I^2^ = 81.1%) (**Table 1** **,** **Figure 5** **,** **6**). Multiple HPV infections were seen in 12.6% (CI 8.7–17.2%; I^2^ = 87.8%) of the samples.

**Figure 6:**
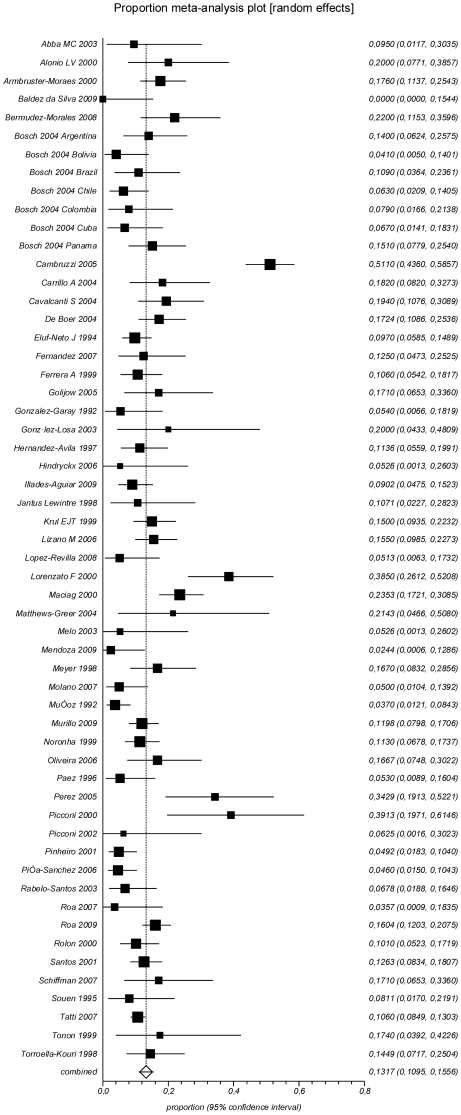
Prevalence of HPV18 in ICC.

**Table 2** shows the prevalence of HPV16/18 in ICC and HSIL by country, region, and GNI World Bank Classification. In Argentina (10 studies), the pooled prevalence of HPV16 in cancer samples was 59.5% (95% CI 51.3–67.5%; I^2^ = 68.2%), in Brazil (13 studies) 53.2% (95% CI 42.9–63.3%; I^2^ = 92.5%), and in Mexico (14 studies) 54.9% (95% CI 47.6–61.9%; I^2^ = 80.3%).

**Figure 7:**
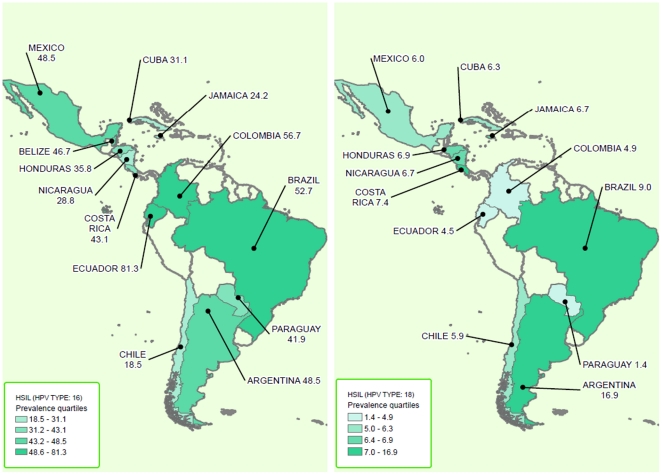
Distribution HPV 16/18 in HSIL in LA&C.

When we analyzed the prevalence of HPV18 in ICC samples, we found a pooled prevalence of 17.6% (95% CI 12–24.1%; I^2^ = 65.7%) in Argentina, 15.8% (95% CI 8.9–24.2%; I^2^ = 92.8%) in Brazil, and 12.8% (95% CI 9.7–16.2%; I^2^ = 47.6%) in Mexico. HPV prevalence by the geographic region and by GNI World Bank Classification are shown in **Table 2**. The analyses by primers used and by tissue source are shown in **Table 3**. The distributions of HPV 16/18 in HSIL and ICC in LA&C according to quartiles of prevalence are shown in maps in **Figures 7** **and** **8**.

**Figure 8:**
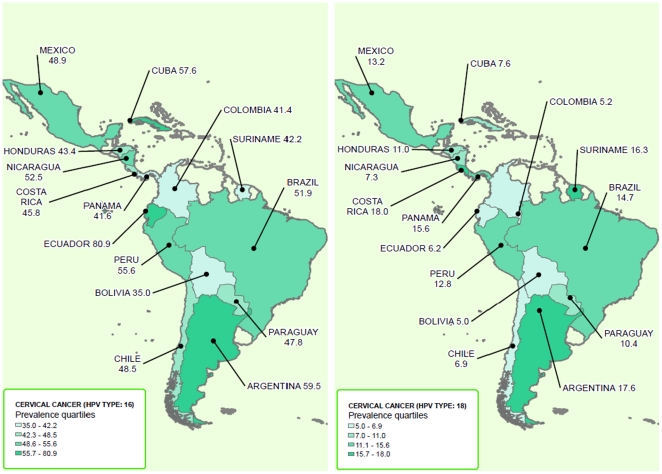
Distribution HPV 16/18 in ICC in LA&C.

We also applied a meta-regression analysis adjusting by GNI World Bank Classification, Geographic region, genotyping method and HPV tissue source to obtain adjusted estimates. There were no statistically significant differences for HPV16 in cancer and in HSIL. For HPV18, the statistically significant difference were seen for HSIL when the tissue source was fixed biopsies (compared to exfoliated cells) and when MY and GP performed together were used compared to Hybridization techniques and for cancer when the tissue source were Polymerase Chain Reaction SPF, GP5/6, E6, E7 and others compared to Hybridization techniques. However the adjusted prevalence, by the means of each variable and considering the SE of the meta-analysis, remained stable: HPV16 in HSIL women 45.7% (CI 95% 42.9–48.5%) HPV18 in HSIL 8.7% (7.2–10.3%); HPV16 in ICC 55.3% (52.5–58.1%); and HPV18 in ICC 13.4 (11.5–15.3).

Funnel plots showed no evidence of publication bias (data not shown).

## Discussion

Data on the geographic distribution of HPV type in HSIL and ICC are crucial for estimating the impact of HPV vaccines on cervical cancer and cervical screening programs. [106], [107] Epidemiological studies employing a variety of HPV typing protocols have been aggregated in some meta-analyses. However, the number of samples from LA&C considered in these studies was relatively low.

This review brings representative estimations of HPV type distribution from the LA&C region. Since multiple HPV genotyping techniques have been included, varying sensitivities of the techniques considered might impact the HPV type-specific prevalence reported [108]. Currently, identification of specific HPV types in biological specimens is preferentially done by PCR-based methods due to its higher sensitivity; in this study, however, hybridization techniques without PCR amplification (membrane and in situ hybridization) were also included in order to incorporate the largest number of HSIL and ICC cases, and to increase the representativeness of the data. Nevertheless, only 6% of studies -the oldest ones- used non-PCR-based techniques.

In 2003, Smith et al. [14] updated a meta-analysis of over 10,000 cases published [10], [11]. It retrieved 1,427 cancer cases and 833 HSIL cases from 13 countries in the LA&C region; the prevalence of HPV 16/18 in cervical cancer for South/Central America was 65%. Muñoz et al., in 2004, included 1,084 cervical cancer cases from Central/South America and found an HPV16/18 prevalence of 69%. [13]. Later, Li et al have published a worldwide meta-analysis of HPV type-specific including a total of 30,848 cervical cancers. It included 3,010 cancer cases from 15 countries of LA&C; in this region for 1990–2010, HPV16 and HPV18 were the first and second most common types, respectively (54% and 15% respectively); being the third to eighth most common types HPVs 31, 45, 33, 58, 52 and 35. [12]. The present systematic tripled the number HSIL cases included in the previous reports of Clifford et al. [10], [11] and Smith et al. [14]. Overall, 55% of HSIL cases harbored HPV 16/18, confirming that HPV type distribution in HSIL does not entirely match that of ICC. HPV types 16, 18 and 45 are less common in HSIL than in ICC, whereas other HPV types are more frequent (particularly, HPV58, the third-most prevalent type in HSIL). These differences emphasize the importance of HPV type in the risk of progression to cancer, even from HSIL. The proportions of HSIL cases attributable to both HPV16 and HPV 18 in this study were higher than those in previous meta-analyses [11],[14], which estimated 48% for the region. Our prevalence HPV 16/18 rate is similar to Europe (57.6%) and North America (55.1%), according to the study published by Smith et al. [14]

Data on ICC has greatly enriched previous reports; we increased the number of Latin American cases included from 3,010 considered by the last published meta-analysis [14] to 5,542 in our study. Regarding ICC cases, 53.2% harbored HPV 16 and 13.2% HPV18, confirming that they are the first- and second-most prevalent types, respectively, which agrees with data previously obtained on other continents and worldwide. The next five-most common types, (HPV 31, 58, 33, 45, and 52) added 22.6% of cases. The proportions of cases attributable to HPV16/18 in this study were similar to previous meta-analyses [10], [11], [14], which estimated nearly 65% for the region. Our findings corroborate that in LA&C the HPV16/18 prevalence of ICC is similar to that of Asia (66.9%) and lower than that of Africa (70%), Europe (73.8%) and North America (76.4%), according Smith et al. [14]

Some intra-regional variations of the most common HPV types have been observed, although these apparent differences may happen simply by random fluctuation and/or a lack of sample representativeness of certain countries. For ICC, Mexico, Central America and the Caribbean showed a slightly lower HPV16/18 prevalence than South America (64.2% vs. 67.3% respectively). Particularly, Argentina shows the highest prevalence rate for HPV16/18 in both HSIL (65.4%) and ICC (77.1%). It is interesting to point out that the 12 Argentine studies incorporated samples from women of different provinces of the country, including aboriginal communities (Quechua [37] and Guarani [39] populations), revealing similar HPV16/18 prevalence data.

In 11.6% of HSIL and 7.5% of ICC, HPV detection resulted positive, but the viral type could not be identified (“other type”); these cases most likely represent the failed detection of known types (almost certainly different than HPV 16 and 18) rather than infections of yet-undiscovered types.

In this review, multiple-type HPV infections were detected in 16.8% of HSIL and 12.6% of ICC, although the frequency of multiple infections depends largely on the number of HPV types tested for within a given study. The attribution of ICC etiology to HPV types is increasingly complicated by the rising prevalence of multiple co-existing types. It was suggested that infections with multiple HPV types seem to act synergistically in cervical carcinogenesis [109], and it was also associated with poor response and with reduced survival in cervical cancer patients. [110]. However, other study indicates that despite the presence of many viruses infecting the same anatomical site, only one genotype would be responsible for the disease [111].

HPV18 and 16 had the highest ICC∶HSIL prevalence ratio in our studies, as found in Smith et al. meta-analysis [14]. Conversely, HPV11, 56, 6, 68 and 58, were each 2 to 3-fold more prevalent in HSIL than in ICC. These lowest ratios were observed for many different types and lower than reported [14].

As more data is accumulated, it is supportive to observe that HPV16/18 accounts for two-thirds of ICC in LA&C. The proportion of ICC cases potentially averted by the current approved vaccines may be even higher than the aforementioned one if cross-protection against non-vaccine high-risk HPV types (like HPV31 and 45) is found to be clinically effective in reducing the incidence of ICC and HSIL caused by these genotypes. The information given by this work would be also useful in LA&C for the evaluation of polyvalent vaccines (currently in development) for the prevention of ICC associated to more than eight or nine high-risk HPV types.

Limitations of our meta-analysis include the cross-sectional design of the included studies and their inherent risk of bias, lack of representativeness, the HPV type-specific prevalence variation and HPV type-specific sensitivity of different PCR protocols [112]. There is evidence of considerable heterogeneity between studies. Heterogeneity could not be ruled out even by the pre-designed subgroup analysis: by country, region, and GNI World Bank classification. However inconsistencies might be explained by variations in the population and methods utilized. To address this issue we chose the random effect model meta-analysis to combine data in order to obtain conservative (wider) confidence intervals, which may result more informative than central estimates. In addition 61% of the patients included in the meta-analysis came from only three countries (Argentina, Brazil and Mexico) and one should be cautious when extrapolating our summary results to the entire region. Further, many studies did not type for a broad range of HPV types, and cyto-histological diagnoses across studies were not standardized. The poor infrastructure of research in molecular biology in many countries highlights the need to consider strategic alliances and promoting regional research consortia on the topic of HPV. In this way, according to the World Health Organization HPV Laboratory Network (WHO HPV LabNet) guidelines, the establishment of a Regional HPV LabNet would be extremely useful [113]. This is initiative would support the laboratory standardization and quality assurance of HPV typing methods to promote international comparability of results, promoting an appropriate vaccine introduction and virological surveillance in the vaccine era.

Although information on the histological type of ICC was collected, its discrimination was not always clear and the data came mostly from SCC. For this reason we presented only global data of ICC.

This study is the broadest summary of HPV type distribution in HSIL and ICC in LA&C to date, and it has included the majority of American countries which have the highest cervical cancer burdens in the region and worldwide. The presented information may be of importance for local decision makers to consider the cervical cancer prevention as a whole, taking into account the relevance of vaccination and updating screening strategies using type-specific high-risk HPV-DNA-based tests. This work comes available at the time some Latin American and Caribbean countries are evaluating the HPV vaccine introduction in their National Vaccination Schedules, in the frame of the Pan American Health Organization purchase using revolving fund, which makes vaccines affordable. Continued surveillance of HPV types in HSIL and ICC as HPV vaccines are introduced would be useful, to assess the potential for changing type-specific HPV prevalence in the post-vaccination era in Latin America.

## Supporting Information

Appendix S1 **Search Strategy.**
(DOC)

Appendix S2 **Methodological Quality Assessment.**
(DOC)

Appendix S3 **Study characteristics and HPV-specific prevalence by study and country.**
(DOC)

Checklist S1 **PRISMA checklist for reporting systematic reviews and meta-analyses.**
(DOC)

Diagram S1 **PRISMA study flow diagram for reporting systematic reviews and meta-analyse.**
(DOC)
