# Supplementary material for: Type-Specific HPV Prevalence in Cervical Cancer and High-Grade Lesions in Latin America and the Caribbean: Systematic Review and Meta-Analysis
Source: PLoS One. 2011 Oct 4;6(10):e25493. doi: 10.1371/journal.pone.0025493 (PMC3186785; doi:10.1371/journal.pone.0025493)
Supplement: Appendix S1 — Search Strategy. (DOC) [file pone.0025493.s001.doc]

**Appendix 1. Search strategy**

**Medline- PubMed search strategy**

(((Uterine Cervical Dysplasia[Mesh] OR Uterine Cervical Neoplasms[Mesh] OR HSIL*[tiab] OR “high grade squamous intraepithelial”[tiab] OR cancer*[tiab] OR tumor[tiab] OR tumors[tiab] OR tumoral*[tiab] OR neoplas*[tiab] OR carcino*[tiab] OR Adenosquam*[tiab] ) **AND** (Cervical[tiab] OR cervix[tiab] OR uter*[tiab])) **AND** (Papillomaviridae[Mesh]OR Alphapapilloma*[tiab] OR Betapapilloma*[tiab] OR Gammapapilloma*[tiab] OR Mupapilloma*[tiab]OR Papilloma*[tiab] OR HPV*[tiab])) OR Papillomavirus Vaccines[Mesh])

**AND** ((Female[Mesh] OR Female*[tiab] OR Women[Mesh] OR Woman[tiab] OR Women[tiab])) **AND** ((Humans[Mesh])) **AND** (Americas[MeSH Terms:noexp] OR America*[all] OR Latin America[Mesh] OR Latin America*[all] OR Latinamerica*[all] OR Latinoamerica*[all] OR Latin*[all] OR Hispanic Americans[Mesh] OR Hispanic America*[all] OR Hispanoamerica*[all] OR Hispano*[all] OR Hispanic*[all] OR Iberoamerica*[all] OR Ibero Americ*[all] OR Panamerican*[all] OR Central America[Mesh] OR Central America*[all] OR Centroamerica*[all] OR Mesoamerica*[all] OR Meso America*[all] OR Middle America*[all] OR South America[Mesh] OR South America*[all] OR Southamerica*[all] OR Sudamerica*[all] OR America del sur[all] OR Caribbean Region[Mesh] OR Caribbean[all] OR Caribe*[all] OR OR West Indies[Mesh] OR West Indi*[all] OR Antill*[all] OR American Native Continental Ancestry Group[Mesh] OR Amerindian*[all] OR Indians[all] OR American Indian*[all] OR Native America*[all] OR Patagoni*[all] OR Andes[all] OR Andean*[all] OR Amazon*[all] OR Argentina[Mesh] OR Argentin*[ad] OR Argentin*[all] OR Argentina[pl] OR Bolivia[Mesh] OR Bolivia*[ad] OR Bolivia*[all] OR Bolivia[pl] OR Brazil[Mesh] OR Brazil*[ad] OR Brasil*[ad] OR Brazil*[all] OR Brasil*[all] OR Brazil[pl] OR Colombia[Mesh] OR Colombia*[ad] OR Colombia*[all] OR Colombia[pl] OR Chile[Mesh] OR Chile*[ad] OR Chile*[all] OR Chile[pl] OR Ecuador[Mesh] OR Ecuador*[ad] OR Ecuator*[ad] OR Ecuador*[all] OR Ecuador[pl] OR French Guiana[Mesh] OR Guiana*[ad] OR Guiana*[all] OR French Guiana[pl] OR Guyana[Mesh] OR Guyan*[ad] OR Guyan*[all] OR Guyana[pl] OR Paraguay[Mesh] OR Paraguay*[ad] OR Paraguay*[all] OR Paraguay[pl] OR Peru[Mesh] OR Peru*[ad] OR Peru*[all] OR Peru[pl] OR Suriname[Mesh] OR Surinam*[ad] OR Surinam*[all] OR Suriname[pl] OR Uruguay[Mesh] OR Uruguay*[ad] OR Uruguay*[all] OR Uruguay[pl] OR Venezuela[Mesh] OR Venez*[ad] OR Venez*[all] OR Venezuela[pl] OR Belize[Mesh] OR Belize*[ad] OR Belize*[all] OR Belize[pl] OR Costa Rica[Mesh] OR Costa Ric*[ad] OR Costarric*[ad] OR Costaric*[ad] OR Costa Ric*[all] OR Costarric*[all] OR Costaric*[all] Costa Rica[pl] OR El Salvador[Mesh] OR Salvador*[ad] OR Salvador*[all] OR El Salvador[pl] OR Guatemala[Mesh] OR Guatemal*[ad] OR Guatemal*[all] OR Guatemala[pl] OR Honduras[Mesh] OR Hondur*[ad] OR Hondur*[all] OR Honduras[pl] OR Nicaragua[Mesh] OR Nicaragu*[ad] OR Nicaragu*[all] OR Nicaragua[pl] OR Panama[Mesh] OR Panam*[ad] OR Panam*[all] OR Panama[pl] OR Mexico[Mesh] OR Mexic*[ad] OR Mexic*[all] OR Mejic*[all] OR Mexico[pl] OR Cuba[Mesh] OR Cuba*[ad] OR Cuba*[all] OR Cuba[pl] OR Dominican Republic[Mesh] OR Dominic*[ad] OR Dominic*[all] OR Dominican Republic[pl] OR Haiti[Mesh] OR Haiti*[ad] OR Haiti*[all] OR Haiti[pl] OR Jamaica[Mesh] OR Jamaic*[ad] OR Jamaic*[all] OR Jamaica[pl])

**LILACS**

(MH Displasia del Cuello del Útero OR MH Neoplasias del Cuello Uterino OR HSIL OR CIN OR (high AND grade AND squamous AND intraepithelial) OR (les$ AND intra$ AND escamosa$ AND alto AND grau) OR (Les$ AND intra$ AND escamosa AND alto AND grau) OR (lesion$ intra$ AND escamosa$ AND alto AND grado) OR cancer OR tumor$ OR neoplasm$ OR carcino$ OR adenosquam$ OR adenoescam$) AND ((MH Papillomaviridae OR Alphapapil$ OR alfapapil$ OR betapapil$ OR gammapapil$ OR gamapapil$ OR mupapil$ OR papilloma$ OR HPV) OR (MH Vacunas contra Papillomavirus)) AND (MH Mujeres OR mujer$ OR Mulher$ OR women OR woman OR MH Femenino OR Feminino$ OR Femenino$ OR female) AND (MH Humanos OR human$)

**EMBASE**

1 exp uterine cervix tumor/ (64683)

2 (cancer$ or tumor$ or neoplas$ or carcino$ or adenosquam$).ti,ab. (1867509)

3 or/1-2 (1881019)

4 (cervix or cervical).ti,ab. (168490)

5 3 and 4 (72542)

6 exp Papilloma virus/ (30291)

7 (Alphapapilloma$ or Betapapilloma$ or Gammapapilloma$ or Mupapilloma$ or Papilloma$ or HPV$).ti,ab. (40991)

8 exp Wart virus vaccine/ (3969)

9 or/6-8 (47277)

10 5 and 9 (14045)

11 exp female/ or exp women/ (4960291)

12 (female$ or women or woman).ti,ab. (1224182)

13 11 or 12 (5177135)

14 exp Human/ or human$.ti,ab. (12741670)

15 10 and 13 and 14 (10472)

16 exp "South and Central America"/ or latin america$.mp. or latinameric$.mp. or south america$.mp. or southamerica$.mp. or central america$.mp. or centroamerica$.mp. or mesoamerica$.mp. or caribbe$.mp. or caribe$.mp. or hispanoamerica$.mp. or hispano america$.mp. or hispanic$.mp. or iberoamerica$.mp. or ibero america$.mp. or exp AMERICAN INDIAN/ or indians.mp. or amerind$.mp. or exp ARGENTINA/ or argentin$.mp. or exp BOLIVIA/ or bolivia$.mp. or exp BRAZIL/ or brazil$.mp. or brasil$.mp. or exp CHILE/ or chile$.mp. or exp COLOMBIA/ or colombia$.mp. or exp ECUADOR/ or ecuador$.mp. or exp French Guiana/ or guiana$.mp. or exp GUYANA/ or guyan$.mp. or exp PARAGUAY/ or paraguay$.mp. or exp PERU/ or peru$.mp. or exp SURINAME/ or surinam$.mp. or exp URUGUAY/ or uruguay$.mp. or exp VENEZUELA/ or venezuel$.mp. or amazon$.mp. or andes.mp. or andean$.mp. or patagoni$.mp. or exp BELIZE/ or belize$.mp. or exp Costa Rica/ or costa ric$.mp. or costaric$.mp. or costarric$.mp. or exp El Salvador/ or salvador$.mp. or exp GUATEMALA/ or guatemal$.mp. or exp HONDURAS/ or hondur$.mp. or exp NICARAGUA/ or nicaragu$.mp. or exp PANAMA/ or paname$.mp. or panama$.mp. or exp MEXICO/ or mexic$.mp. or antill$.mp. or exp CUBA/ or cuba$.mp. or exp Dominican Republic/ or dominic$.mp. or exp HAITI/ or haiti$.mp. or exp JAMAICA/ or jamaic$.mp. or exp Caribbean Islands/ or west indies.mp. or exp Puerto Rico/ or Puerto Ric$.mp. or Puertoric$.mp. (260720)

17 15 and 16 (692)
