# Supplementary material for: Type-Specific HPV Prevalence in Cervical Cancer and High-Grade Lesions in Latin America and the Caribbean: Systematic Review and Meta-Analysis
Source: PLoS One. 2011 Oct 4;6(10):e25493. doi: 10.1371/journal.pone.0025493 (PMC3186785; doi:10.1371/journal.pone.0025493)
Supplement: Appendix S2 — Methodological Quality Assessment. (DOC) [file pone.0025493.s002.doc]

**Appendix 2. Methodological Quality Assessment By Study Design**

Tool for assessing susceptibility to bias in observational studies

The risk of bias of observational studies should be assessed by pairs of independent researchers through a checklist of essential items stated in STROBE^1^ (Strengthening the Reporting of Observational studies in Epidemiology), and considering two methodological articles: Sanderson et al.^2^ and Fowkeset al.^3^, QATSO ^4^, Berra ^5^. We propose an algorithm, programmed in an Excel spreadsheet, to estimate a summary risk of bias considering three four criteria (methods for selecting study participants, methods for measuring exposure and outcome variables, methods to control confounding, and comparability among groups.) and two minor criteria (statistical methods excluding confounding, and conflict of interest). Disagreements should be solved by consensus.

| **Domain** | **Tool item must address** | **Met domain criteria?#** | | | | |
| --- | --- | --- | --- | --- | --- | --- |
|  |  | **N** | **P** | **Y** | **U** | **NA** |
|  | **MAJOR CRITERIA** |  |  |  |  |  |
| ***Methods for selecting study participants** | The selection procedures (inclusion and exclusion criteria, sources and selection methods) are appropriate for include a sample which is representative of the universe of interest?  Ideally sampling should be probabilistic (all the participants have the same probability of be selected) random or have used an appropriate method to avoid selection bias and population based.  The risk of bias is high in convenience samples or if the technique of sampling or selection of controls is not clear. |  |  |  |  |  |
|  | The sample included in the study, has on it a representative spectrum of the population of interest?  Take into account how the study sample is representative of the population of interest (general population, admitted patients, outpatients in a health center, age group, schooled). The risk of bias increases in studies conducted in special populations. In case-control studies, cases should represent all the cases and controls represent the community from which they come.  Notice if the specific groups within the sample (level of instruction, employment, origin, etc) are proportionally represented.  Assess if the cases and the controls come for the same population and if they come of a consecutive series or if there is any kind of selection involved. |  |  |  |  |  |
|  | An estimation of the sample size has been made?  Assess if the estimation has been made for employment, place of residence, level of instruction |  |  |  |  |  |
|  | The flow of persons potentially illegible, initially selected, the ones who accepted to participate and the ones who actually participate affect the extrapolation of results to the population of interest?  Analyze differences and similarities between the population of interest and the studied one taking into account the spatial and temporal context (e.g. prevalence of exposition), inclusion criteria, definition and measurement of the exposition and the outcome, confidence on the estimations, etc. |  |  |  |  |  |
|  | ***Summarizing, an adequate sample, similar to the population of interest minimizes the selection bias*** |  |  |  |  |  |
| ***Methods for measuring exposure and outcome variables: intervention /exposition, outcome, confounding o modifier variable**  (In comparative studies the methods for measuring must be the same for both groups) | The principal variables have an adequate conceptual (theoretical) and operational (measurement scale classification system, diagnostic criteria, etc.) definitions?  Clear definition of the cases and the controls. The state of illness of the patients in the cases group has been determined and validated in a reliable way. |  |  |  |  |  |
|  | The instruments used to measure the principal variables have validity and reliability which are known and adequate?  Assess if studies which evaluated these instruments are cited. To questionnaires originally designed for a determined culture or language, if they have been adapted to where there are going to be used. Notice whether the data were obtained from direct measurements, survey of medical records or self report events. Take into account if inter and intra observer variability has been reported. |  |  |  |  |  |
|  | The techniques used to measure the principal variables are adequately described? Are appropriate? In case of comparative studies, are the same for both groups?  Take into consideration the possibility of recall bias (the cases remember better the expositions than the controls) or interviewer bias. If the interviewer training was necessary, noted if it has been done. |  |  |  |  |  |
|  | Are there co-interventions or co-exposures that might have biased the results? |  |  |  |  |  |
|  | The results were evaluated blindly or through objective criteria? |  |  |  |  |  |
|  | The follow up was long enough to allow observe the outcomes? |  |  |  |  |  |
|  | Was there quality control of the primary data? |  |  |  |  |  |
|  | ***Summarizing, the measurement and survey of the principal variables have been made in an appropriate way. The risk of information bias has been minimized*** |  |  |  |  |  |
| ***Methods for confounders control** | Have been taken into account the potential main confounders in the design and analysis of the study? In the design, variables associated with the studied problem should be taken into account; and in the analysis, estimation of the primary outcome should be stratified or adjusted by these variables (multivariate analysis).  Notice if raw and adjusted measures have been reported, if the variables by which the results were adjusted are indicated, and if justification of which included (or not) in the analysis. Consider whether there were deviations in the protocol and the reasons of why. |  |  |  |  |  |
|  | ***Summarizing, all known confounding variables have been gathered and the analysis has been adjusted by them.*** |  |  |  |  |  |
| ***Comparability among groups.**  If there is no comparison between groups, the answer must be “not applicable” in each statement in this category | Are the source populations of the individuals in each group and the periods of recruitment similar? (sociodemographic variables, prognostic factors or other variables who might alter the results).  According to the selection process, both populations have similar characteristics, are comparable throughout except for the study factors (cases and controls definition).  In cohort studies should assess whether patients were recruited at a similar point in the evolution of the disease. |  |  |  |  |  |
|  | Were used the same strategies and measurement techniques in both groups? Were measured the same variables? |  |  |  |  |  |
|  | The losses (missing data, dropouts, emigration, etc) affect the groups in different ways?  The differences between groups concerning the losses must be less than 20%. Non-response rate and the reasons of non response are similar. A high non-response rate could be accepted if there is evidence that the groups remain similar. In case-control studies assess if there overmatching has been existed. |  |  |  |  |  |
|  | ***Summarizing, the groups are comparable; the possibility of selection bias has been minimized.*** |  |  |  |  |  |
|  | **MINOR CRITERIA** |  |  |  |  |  |
| **Statistical Methods** (excluding control of confounding) | The statistical analysis was determined at the onset of the study? (not post-hoc). |  |  |  |  |  |
|  | The statistical tests are appropriate? The allocation unit (community, institution, group, cluster, individuals) and the unit of analysis match? In case of mismatch an adjustment has been made? Are the methods appropriate to any anomalous data distributions (eg skewed, multimodal?) Was there adequate adjustment for any differences in length of follow-up (prospective studies) or time between the intervention and outcome (case-controls)? |  |  |  |  |  |
|  | In the analysis, losses of participants, missing data or other design defects (e.g. different probabilities of selection) were adequately treated?  In certain cases the exclusion of cases to allow perform sensitivity analysis would be appropriate.  An intention to treat analysis would be suitable for prospective comparative studies.  Losses of more than 20% in short follow-up (less than a year) and more than 30% in larger periods should be considered high. In any case losses shouldn’t affect the minimum sample size required to perform the statistical analysis. |  |  |  |  |  |
|  | Does reporting address parameter estimate uncertainty (ie with SEs or CIs)? |  |  |  |  |  |
|  | Was there a dose-response relationship between the intervention/exposition and the outcome shown? |  |  |  |  |  |
|  | ***Summarizing, the analysis is appropriate.*** |  |  |  |  |  |
| **Conflict of interest** | ***Summarizing, the conflict of interest does not condition the results or the conclusions of the study.*** |  |  |  |  |  |

**DOMAIN VALUATION** (must consider only the applicable items) **N** (**No**) **P** (**Partially**) **Y** (**Yes**)

**U** (Unclear accomplishment of domain criteria because there is no enough information to assess) should be considered **N** for major criteria and **P** for minor criteria.

**NA** (**N**ot **A**pplicable because of the design)

**OVERALL RISK OF BIAS ASSESSMENT**

| **HIGH** | ≥ 1 criteria (major or minor*)= N; or  ≥ 1 major criteria =U; o  ≥2 major criteria =P |
| --- | --- |
| **MODERATE** | Both minor criteria= P o U  1 major criteria =P and 1 minor criteria= P o U =P  None or 1 major criteria =M and the rest Y |
| **LOW** | All the major criteria= Y and none or 1 minor criteria= P o U |

* justify

In case there were items qualified as **NA** (**N**ot **A**pplicable because of the design) and the criteria for any risk of bias were no met, the summay risk of bias will be decided on the basis of most of the available criteria.

1 criteria P between the Applicable majors = **M** (**M**oderate risk of bias)

E.g. 3 criteria applicable P, Y, P = **M** (**M**oderate risk of bias)

E.g. 3 criteria applicable Y, P, Y =  **M** (**M**oderate risk of bias)

If the selection is P, Y, Y = **M** (**M**oderate risk of bias)

E.g. 2 criteria applicable P, Y = **M** (**M**oderate risk of bias)

If none major criteria are applicable the study has a risk of bias Not Applicable.

This tool is currently being validated.

**References**

1. von Elm E, Altman DG, Egger M, Pocock SJ, Gotzsche PC, Vandenbroucke JP. The Strengthening the Reporting of Observational Studies in Epidemiology (STROBE) statement: guidelines for reporting observational studies. Lancet 2007;370:1453-7.

2. Sanderson S, Tatt I, Higgins J. Tools for assessing quality and susceptibility to bias in observational studies in epidemiology: a systematic review and annotated bibliography

2007;36:666-76.

3. Fowkes F, Fulton P. Critical appraisal of published research: introductory guidelines. BMJ 1991 302:1136-40.

4. Wong WC, Cheung CS, Hart GJ. Development of a quality assessment tool for systematic reviews of observational studies (QATSO) of HIV prevalence in men having sex with men and associated risk behaviours. Emerg Themes Epidemiol 2008;5:23.

5. Berra S, Elorza-Ricart JM, Estrada M-D, Sánchez E. Instrumento para la lectura crítica y la evaluación de estudios epidemiológicos transversales. Gaceta Sanitaria 2008;22:492-7.
