# Supplementary material for: Type-Specific HPV Prevalence in Cervical Cancer and High-Grade Lesions in Latin America and the Caribbean: Systematic Review and Meta-Analysis
Source: PLoS One. 2011 Oct 4;6(10):e25493. doi: 10.1371/journal.pone.0025493 (PMC3186785; doi:10.1371/journal.pone.0025493)
Supplement: Appendix S3 — Study characteristics and HPV-specific prevalence by study and country. (DOC) [file pone.0025493.s003.doc]

**APPENDIX I-** **Characteristics and HPV-specific prevalence among women with HSIL/cervical cancer, by study and country**

| **Country**  (S)N [%] | **Study** | **Design** | | **Bias Risk** | | **Age** | **Age**  **range** | **Hpv**  **source** | **Primer** | **Cervical**  **Lesion** | **N** | **HPV-specific prevalence (% of all cases tested)** | | | | | | | | | | | | | | | | | | | | | | | | |
| --- | --- | --- | --- | --- | --- | --- | --- | --- | --- | --- | --- | --- | --- | --- | --- | --- | --- | --- | --- | --- | --- | --- | --- | --- | --- | --- | --- | --- | --- | --- | --- | --- | --- | --- | --- | --- |
|  |  |  |  |  |  |  |  |  |  |  |  | **Any** | **16** | | **18** | **45** | **31** | | **33** | | **58** | | **52** | **35** | **59** | | | **56** | **51** | **68** | **39** | **66** | **6** | **11** | **Other** | **Mul** |
| **Argentina**  (15)  1515  [19.0] | Abba 2003^[1]^ | C-S | | H | |  |  | Cells | MY+GP | HSIL | 82 | 97.6 | 48.8 | | 13.4 |  | 7.3 | | 2.4 | |  | |  |  |  | | |  | 7.3 |  |  |  | 15.9 | 12.2 |  | 13.7 |
|  |  |  |  |  |  |  |  |  |  | ICC | 21 | 100.0 | 66.6 | | 9.5 |  | 4.7 | |  | |  | |  |  |  | | |  | 19.0 |  |  |  | 14.3 | 12.2 |  | 14.3 |
|  | Alonio 1990^[2]^ | C-C | | H | |  | 28-40 | Fixed Bio. | ISH | HSIL | 61 | 60.7 | 26.2 | | 34.4 |  |  | |  | |  | |  |  |  | | |  |  |  |  |  | 14.8 | 14.8 |  | 29.5 |
|  | Alonio 2000^[3]^ | C-S | | H | |  | 27-48 | Fixed Bio. | GP5/6 | HSIL | 36 | 80.6 | 41.7 | | 11.1 | 0.0 | 0.0 | | 5.6 | | 0.0 | | 0.0 | 0.0 | 0.0 | | | 0.0 | 0.0 | 0.0 | 0.0 | 0.0 | 0.0 | 0.0 | 22.2 | 0.0 |
|  |  |  |  |  |  |  | 38-56 |  |  | ICC | 30 | 93.3 | 46.7 | | 20.0 | 0.0 | 3.3 | | 3.3 | | 0.0 | | 0.0 | 0.0 | 0.0 | | | 0.0 | 0.0 | 0.0 | 0.0 | 0.0 | 6.7 | 0.0 | 13.3 | 6.7 |
|  | Alonio 2003^[4]^ | C-S | | H | | 38.2 |  | Fresh Bio. | GP5/6 | HSIL | 50 | 84.0 | 54.0 | | 12.0 |  | 0.0 | | 10.0 | |  | |  |  |  | | |  |  |  |  |  | 0.0 | 0.0 | 8.0 |  |
|  | Bosch (2004) 1995^[5]*^ | C-S | | M | |  |  | Fixed bio. | ISH | ICC | 57 | 98.2 | 61.4 | | 14.0 | 5.3 | 5.3 | | 1.8 | | 1.8 | | 1.8 | 0.0 | 1.8 | | | 0.0 | 0.0 | 0.0 | 3.5 | 0.0 | 0.0 | 0.0 | 1.8 |  |
|  | Chouhy 2006^[6]^ | C-S | | H | | 30.6 | 18-68 | Cell+Fresh | MY/C | HSIL | 12 | 83.0 | 58.0 | | 0.0 | 0.0 | 8.0 | | 17.0 | | 0.0 | | 0.0 | 0.0 | 0.0 | | | 0.0 | 0.0 | 0.0 | 0.0 | 0.0 | 0.0 | 0.0 | 0.0 | 0.0 |
|  | Deluca 2002^[7]^ | C-S | | H | | 31.0 | 18-55 | Cells | MY/C | HSIL | 12 | 83.3 | 25.0 | |  |  |  | |  | | 8.3 | |  |  |  | | |  |  |  |  |  |  |  | 16.7 |  |
|  | Deluca 2006^[8]^ | C-S | | H | | 31.0 | 18-55 | Cells | MY/C | HSIL | 14 | 85.7 | 21.4 | | 7.1 | 0.0 | 14.3 | | 0.0 | | 21.4 | | 0.0 | 0.0 | 0.0 | | | 0.0 | 0.0 | 0.0 | 0.0 | 0.0 | 7.1 | 0.0 | 14.3 | 0.0 |
|  | Golijow 2005^[9]^ | C-S | | H | | 33.0 | 15-62 | Cells | MY+GP | HSIL | 75 | 98.7 | 56.0 | | 20.0 |  | 5.3 | | 4.0 | |  | |  |  |  | | |  | 12.0 |  |  |  | 22.7 | 6.7 |  | 28.0 |
|  |  |  |  |  |  | 46.0 | 21-66 |  |  | ICC | 35 | 100.0 | 68.6 | | 17.1 |  | 5.7 | | 2.9 | |  | |  |  |  | | |  | 14.3 |  |  |  | 8.6 | 0.0 |  | 17.1 |
|  | Jantus Lewintre 1998^[10]^ | C-C | | H | | 41.0 | 13-71 | Cells | MY/C | HSIL | 22 | 81.8 | 31.8 | | 9.1 | 0.0 | 0.0 | | 4.5 | | 0.0 | | 0.0 | 0.0 | 0.0 | | | 0.0 | 0.0 | 0.0 | 0.0 | 0.0 | 0.0 | 0.0 | 36.4 |  |
|  |  |  |  |  |  |  |  |  |  | ICC | 28 | 89.3 | 42.9 | | 10.7 | 0.0 | 0.0 | | 3.6 | | 0.0 | | 0.0 | 0.0 | 0.0 | | | 0.0 | 0.0 | 0.0 | 0.0 | 0.0 | 0.0 | 3.6 | 28.6 |  |
|  | Perez 2005^[11]^ | C-S | | H | |  |  | Fixed Bio. | MY+GP | HSIL | 74 |  | 85.1 | | 48.6 |  |  | |  | |  | |  |  |  | | |  |  |  |  |  |  |  |  | 28.3 |
|  |  |  |  |  |  |  |  |  |  | ICC | 35 |  | 85.7 | | 34.3 |  |  | |  | |  | |  |  |  | | |  |  |  |  |  |  |  |  | 17.1 |
|  | Picconi 2000^[12]^ | C-S | | H | | 50.0 | 45-55 | Fixed Bio. | GP5/6 | ICC | 23 | 95.7 | 39.1 | | 39.1 | 0.0 | 4.3 | | 0.0 | | 0.0 | | 0.0 | 0.0 | 0.0 | | | 0.0 | 0.0 | 0.0 | 0.0 | 0.0 | 0.0 | 0.0 | 13.0 | 0.0 |
|  | Picconi 2002^[13]^ | C-S | | H | | 35.0 | 14-64 | Fixed Bio. | MY/C | HSIL | 35 | 97.0 | 45.7 | | 8.6 | 2.9 | 5.7 | | 2.9 | | 0.0 | | 0.0 | 0.0 | 0.0 | | | 0.0 | 0.0 | 0.0 | 0.0 | 0.0 | 0.0 | 0.0 | 0.0 | 31.0 |
|  |  |  |  |  |  |  |  |  |  | ICC | 16 | 100.0 | 37.5 | | 6.3 | 6.3 | 12.5 | | 6.3 | | 0.0 | | 0.0 | 0.0 | 0.0 | | | 0.0 | 0.0 | 0.0 | 0.0 | 0.0 | 0.0 | 0.0 | 0.0 | 13.0 |
|  | Tatti 2007^[14]^ | C-S | | H | |  |  | Fresh Bio. | SPF | ICC | 750 |  | 64.7 | | 10.6 | 4.9 | 4.7 | | 4.5 | |  | |  |  |  | | |  |  |  |  |  |  |  | 10.6 |  |
|  | Tonon 1999^[15]^ | C-C | | H | | 34.0 | 17-69 | Cells | GP5/6 | HSIL | 29 |  | 72.4 | | 13.8 |  |  | |  | |  | |  |  |  | | |  |  |  |  |  | 27.6 | 13.8 | 3.5 |  |
|  |  |  |  |  |  |  |  |  |  | ICC | 18 |  | 66.6 | | 17.4 |  |  | |  | |  | |  |  |  | | |  |  |  |  |  | 0.0 | 0.0 | 16.0 | 0.0 |
| **Barbados**  (1) 21 [0.3] | Prussia 1993^[16]^ | C-S | | H | | 55.7 | 31-100 | Fixed Bio. | E6/E7/O | ICC | 21 | 90.0 | 71.0 | |  | 6.0 | 6.0 | |  | |  | |  |  |  | | |  |  |  |  |  |  |  |  |  |
| **Belize**  (1) 15 [0.2] | Cathro 2009^[17]^ | C-S | | M | | 39.6 |  | Cell+Fresh | GP5/6 | HSIL | 15 | 24.6 | 11.5 | | 0.0 | 0.0 | 3.3 | | 0.0 | | 4.9 | | 1.6 | 4.9 | 1.6 | | | 0.0 | 0.0 | 0.0 | 0.0 | 0.0 | 0.0 | 0.0 | 1.6 | 8.2 |
| **Bolivia**  (1) 49 [0.6] | Bosch (2004) 1995^[5]*^ | C-S | | M | |  |  | Fixed bio. | ISH | ICC | 49 | 95.9 | 34.7 | | 4.1 | 8.2 | 26.5 | | 0.0 | | 2.0 | | 4.2 | 0.0 | 2.0 | | | 0.0 | 0.0 | 0.0 | 2.0 | 0.0 | 0.0 | 0.0 | 4.1 |  |
| **Brazil**  (21)  1892  [23.7] | Armbruster -Moraes 2000^[18]^ | C-S | | H | |  |  | Cell+Fresh | SB/DB/F | ICC | 125 | 48.0 | 22.4 | | 17.6 | 0.0 | 0.0 | | 4.0 | | 0.0 | | 0.0 | 0.0 | 0.0 | | | 0.0 | 0.0 | 0.0 | 0.0 | 0.0 | 0.0 | 0.0 | 0.0 | 4.0 |
|  | Bagarelli 2004^[20]^ | C-S | | H | |  |  | Fixed Bio. | ISH | HSIL | 33 |  | 36.4 | | 15.2 |  | 9.1 | | 15.2 | |  | |  |  |  | | |  |  |  |  |  | 3.0 | 0.0 | 33.3 |  |
|  | Baldez da Silva 2009^[19]^ | C-S | | H | |  | 18-35 | Cells | MY/C | HSIL | 29 | 100 | 75.9 | | 6.9 |  | 44.8 | | 6.9 | |  | |  |  |  | | |  |  |  |  |  |  |  |  |  |
|  |  |  |  |  |  |  |  |  |  | ICC | 22 | 100 | 90.9 | | 18.2 |  | 13.6 | |  | |  | |  |  |  | | |  |  |  |  |  |  |  |  |  |
|  | Bosch (2004) 1995^[5]*^ | C-S | | M | |  |  | Fixed bio. | ISH | ICC | 46 | 95.7 | 54.3 | | 10.9 | 4.3 | 4.3 | | 4.3 | | 2.2 | | 2.2 | 4.3 | 0.0 | | | 0.0 | 2.2 | 0.0 | 4.3 | 0.0 | 0.0 | 0.0 | 4.3 |  |
|  | Cambruzzi 2005^[21]^ | C-S | | M | | 50.8 | 21-94 | Fixed Bio. | SPF | ICC | 182 |  | 34.1 | | 51.1 |  |  | |  | |  | |  |  |  | | |  |  |  |  |  |  |  | 14.8 |  |
|  | Cavalcanti 1994^[22]^ | C-S | | H | | 37.1 | 13-80 | Fixed Bio. | ISH | HSIL | 36 | 58.3 | 36.1 | | 16.7 |  |  | |  | |  | |  |  |  | | |  |  |  |  |  | 13.9 | 2.8 |  | 27.8 |
|  |  |  |  |  |  |  |  |  |  | ICC | 67 | 41.8 | 22.4 | | 19.4 |  |  | |  | |  | |  |  |  | | |  |  |  |  |  | 11.9 | 1.5 |  | 11.9 |
|  | Cavalcanti 1996^[23]^ | C-S | | H | | 38.7 | 16-75 | Fixed Bio. | MY/C | HSIL | 103 | 78.6 |  | |  |  |  | |  | |  | |  |  |  | | |  |  |  |  |  |  |  |  | 20.4 |
|  |  |  |  |  |  |  |  |  |  | ICC | 54 | 68.5 |  | |  |  |  | |  | |  | |  |  |  | | |  |  |  |  |  |  |  |  | 7.4 |
|  | Eluf-Neto 1994^[24]^ | C-C | | H | |  |  | Cells | GP5/6 | ICC | 186 | 84.4 | 53.8 | | 9.7 |  | 2.2 | | 3.2 | |  | |  |  |  | | |  |  | 0.0 |  |  |  |  |  |  |
|  | Fernandes 2009^[25]^ | C-S | | H | |  | 15-65 | Cells | ISH | HSIL | 58 |  | 75.0 | |  |  |  | |  | | 7.5 | |  |  |  | | |  |  |  |  |  |  |  | 15.0 | 15.0 |
|  | Franco 1999^[26]^ | Cohort | | H | | 32.9 | 18-60 | Cells | MY/C | HSIL | 21 |  | 47.6 | | 4.8 | 0.0 | 9.5 | | 0.0 | | 9.5 | | 4.8 | 0.0 | 0.0 | | | 0.0 | 0.0 | 0.0 | 0.0 | 0.0 | 0.0 | 0.0 | 14.3 |  |
|  | Freitas 2007^[27]^ | C-S | | M | |  |  | Cell+Fresh | MY+GP | HSIL | 10 | 100 | 0.4 | | 0 |  |  | |  | |  | |  |  |  | | |  |  |  |  |  |  |  | 0.5 | 0.1 |
|  | Lorenzato 2000^[28]^ | C-C  nested | | H | | 32.4 | 13-84 | Cells | MY/C | HSIL | 60 | 86.7 | 56.7 | | 3.3 | 3.3 | 3.3 | | 8.3 | | 10.0 | | 0.0 | 1.7 | 0.0 | | | 0.0 | 0.0 | 0.0 | 0.0 | 0.0 | 0.0 | 0.0 |  |  |
|  |  |  |  |  |  |  |  |  |  | ICC | 59 | 89.8 | 59.3 | | 38.5 | 0.0 | 11.9 | | 5.1 | | 3.4 | | 1.7 | 3.4 | 0.0 | | | 0.0 | 0.0 | 0.0 | 0.0 | 0.0 | 0.0 | 0.0 |  |  |
|  | Maciag 2000^[29]^ | C-C | | H | | 52.0 | 16-84 | Cell+Fresh | MY/C | ICC | 161 | 96.7 | 73.2 | | 23.5 | 6.5 | 4.6 | | 8.5 | | 2.0 | | 0.7 | 2.0 |  | | | 0.7 | 1.3 |  |  | 0.7 |  |  | 3.3 | 37.9 |
|  | Noronha 1999^[30]^ | C-S | | H | | 45.6 | 43.6 + | Fixed Bio. | MY/C | HSIL | 54 | 63.0 | 54.5 | | 3.0 |  |  | |  | |  | |  |  |  | | |  |  |  |  |  |  |  | 42.5 |  |
|  |  |  |  |  |  |  | 51.5 + |  |  | ICC | 155 | 70.3 | 60.4 | | 11.3 |  |  | |  | |  | |  |  |  | | |  |  |  |  |  |  |  | 28.3 |  |
|  | Oliveira 2003^[31]^ | C-S | | H | | 37.8 | 18-68 | Fresh Bio. | MY/C | HSIL | 14 | 85.7 | 78.6 | | 14.3 |  |  | | 0.0 | |  | |  |  |  | | |  |  |  |  |  | 7.1 | 0.0 |  | 14.3 |
|  | Oliveira 2006^[32]^ | C-S | | H | | 31.5 | 15-70 | Cells | MY/C | HSIL | 61 | 85.2 | 54.1 | | 16.4 | 3.3 | 0.0 | | 0.0 | | 8.2 | |  | 1.6 |  | | |  |  |  |  |  | 8.2 | 0.0 | 0.0 | 14.8 |
|  |  |  |  |  |  |  |  |  |  | ICC | 48 | 95.8 | 56.3 | | 16.7 | 2.1 | 0.0 | | 4.2 | | 6.3 | |  | 6.3 |  | | |  |  |  |  |  | 14.6 | 4.2 | 0.0 | 31.3 |
|  | Pinheiro 2001^[33]^ | C-S | | H | |  |  | Fresh Bio. | MY/C | ICC | 122 |  | 64.8 | | 4.9 | 1.6 |  | | 1.6 | |  | |  |  |  | | |  |  |  |  |  |  |  |  |  |
|  | Rabelo-Santos 2003^[34]^ | C-S | | H | | 49.1 | 27-83 | Fresh Bio. | E6/E7/O | HSIL | 18 | 61.1 | 50.0 | | 0.0 | 0.0 | 5.6 | | 5.6 | |  | |  |  |  | | |  |  |  |  |  |  |  |  | 0.0 |
|  |  |  |  |  |  |  |  |  |  | ICC | 59 | 76.3 | 54.2 | | 6.8 |  | 1.7 | | 8.5 | |  | |  |  |  | | |  |  |  |  |  |  |  | 6.8 | 0.0 |
|  | Rabelo-Santos 2009^[35]^ | PFU | | M | |  | 18-60 | Cells | MY/C | HSIL | 39 | 89.7 | 35.9 | | 2.6 | 2.6 | 7.7 | | 2.6 | | 12.8 | | 10.3 | 2.6 | 0.0 | | | 0.0 | 0.0 | 0.0 | 0.0 | 0.0 | 0.0 | 0.0 | 0.0 | 12.8 |
|  | Souen 1995^[36]^ | C-S | | H | |  |  | Fixed Bio. | ISH | ICC | 37 | 89.2 | 51.4 | | 8.1 |  | 2.7 | | 5.4 | |  | |  |  |  | | |  |  |  |  |  |  |  | 24.3 | 2.7 |
|  | Terra 2007^[37]^ | C-S | | H | |  |  | Fixed Bio. | GP5/6 | HSIL | 33 |  | 69.7 | | 33.3 |  |  | |  | |  | |  |  |  | | |  |  |  |  |  |  |  |  | 21.2 |
| **Chile**  (5)  515  [6.4] | Aedo 2007^[38]^ | C-S | | H | |  | 18-77 | Cells | MY+GP | HSIL | 44 |  | 34.1 | | 13.6 |  | 2.3 | | 4.5 | | 2.3 | | 9.1 | 2.3 | 9.1 | | | 2.3 |  |  | 4.5 |  |  | 2.3 | 13.6 |  |
|  | Bosch (2004) 1995^[5]*^ | C-S | | M | |  |  | Fixed bio. | ISH | ICC | 80 | 98.8 | 50.0 | | 6.3 | 8.8 | 8.8 | | 7.5 | | 0.0 | | 6.3 | 1.3 | 2.5 | | | 1.3 | 0.0 | 1.3 | 3.8 | 0.0 | 0.0 | 0.0 | 1.3 |  |
|  | Melo 2003^[39]^ | C-S | | H | |  | 18-79 | Fresh bio. | E6/E7/O | HSIL | 29 |  | 10.4 | | 0.0 | 10.4 | 13.8 | | 31.0 | | 0.0 | | 17.2 | 3.4 | 0.0 | | | 0.0 | 0.0 |  | 0.0 |  | 0.0 | 0.0 | 0.0 | 13.8 |
|  |  |  |  |  |  |  |  |  |  | ICC | 19 |  | 42.1 | | 5.3 | 10.5 | 0.0 | | 31.6 | | 0.0 | | 5.3 | 0.0 | 0.0 | | | 0.0 | 0.0 |  | 0.0 |  | 0.0 | 0.0 | 0.0 | 5.3 |
|  | Roa 2007^[40]^ | C-S | | H | | 39 | 24-79 | Fixed bio. | MY/C | HSIL | 22 |  | 9.1 | |  | 9.1 | 9.1 | | 45.5 | |  | | 4.5 | 4.5 |  | | |  |  |  |  |  |  |  |  | 18.2 |
|  |  |  |  |  |  |  |  |  |  | ICC | 28 |  | 32.1 | | 3.6 | 7.1 | 0.0 | | 14.3 | |  | | 3.6 | 0.0 |  | | |  |  |  |  |  |  |  |  |  |
|  | Roa 2009^[41]^ | C-S | | H | |  | 20-60+ | Fixed bio. | GP5/6 | ICC | 293 | 94.2 | 76.5 | | 16.0 | 3.8 | 29.4 | | 2.4 | | 2.4 | | 0.7 | 0.7 | 1.0 | | | 0.7 | 0.0 | 0.0 | 2.0 | 3.1 | 1.0 | 13.7 | 1.0 | 27.0 |
| **Colombia**  (7)  691  [8.7] | Bosch (2004) 1995^[5]*^ | C-S | | M | |  |  | Fixed bio. | ISH | ICC | 38 | 97.4 | 52.6 | | 7.9 | 5.3 | 7.9 | | 2.6 | | 2.6 | | 2.6 | 7.9 | 2.6 | | | 0.0 | 0.0 | 2.6 | 2.6 | 0.0 | 0.0 | 0.0 | 0.0 |  |
|  | Bosch 1993^[42]^ | C-C | | H | | 40.0 | 18-70 | Cells | SB/DB/F | HSIL | 125 | 63.2 | 32.8 | | 0.0 |  | 2.4 | | 2.4 | |  | |  | 1.6 |  | | |  |  |  |  |  | 0.0 | 0.0 | 24.0 |  |
|  | Molano 2007^[43]^ | C-S | | H | |  | 28-65 | Cells | MY/C | ICC | 60 |  | 53.3 | | 5.0 | 1.7 |  | |  | |  | |  |  |  | | |  |  |  |  |  |  |  | 15.0 |  |
|  | Muñoz 1992^[44]^ | C-C | | H | | 46.5 |  | Fresh bio. | E6/E7/O | ICC | 135 | 46.7 | 32.6 | | 3.7 |  | 3.7 | | 0.0 | |  | |  | 2.2 |  | | |  |  |  |  |  | 0.0 | 0.0 |  |  |
|  | Muñoz 2009^[45]^ | Cohort | | M | |  | 15-85 | Cells | E6/E7/O | HSIL | 32 |  | 78.1 | |  |  |  | |  | |  | |  |  |  | | |  |  |  |  |  |  |  |  |  |
|  | Murillo 2009^[46]^ | C-S | | H | | 51.5 |  | Fixed bio. | MY/C | ICC | 217 |  | 51.2 | | 12.0 | 8.3 | 6.0 | | 4.6 | | 5.5 | | 2.3 | 1.8 | 2.8 | | | 3.2 | 2.3 | 1.4 | 1.4 | 0.9 | 0.0 | 0.0 | 18.4 | 16.6 |
|  | Sierra Torres 2006^[47]^ | C-C | | H | | 44.5 |  | Fresh Bio. | E6/E7/O | HSIL | 84 | 92.0 | 61.0 | | 15.0 |  | 7.0 | | 6.0 | |  | |  |  |  | | |  |  |  |  |  |  |  | 3.0 |  |
| **Costa Rica**  (1)165[2.1] | Schiffman (2007) 2005^[48]^ | C-S | | M | | 38.0 | 18-96 | Cells | MY/C | HSIL | 130 | 85.4 | 43.1 | | 6.9 | 4.6 | 9.2 | | 4.6 | | 13.1 | | 7.7 | 3.1 | 1.5 | | | 2.3 | 7.7 | 0.8 | 4.6 | 1.5 | 1.5 | 1.5 | 33.5 | 33.9 |
|  |  |  |  |  |  |  |  |  |  | ICC | 35 | 77.1 | 45.7 | | 17.1 | 5.7 | 5.7 | | 8.6 | | 11.4 | | 8.6 | 0.0 | 0.0 | | | 0.0 | 2.9 | 0.0 | 2.9 | 2.9 | 0.0 | 2.9 | 11.4 | 31.4 |
| **Cuba**  (2) 90 [1.1] | Bosch (2004) 1995^[5]*^ | C-S | | M | |  |  | Fixed bio. | ISH | ICC | 45 | 97.8 | 57.8 | | 6.7 | 6.7 | 6.7 | | 0.0 | | 0.0 | | 2.2 | 0.0 | 2.2 | | | 2.2 | 2.2 | 0.0 | 4.4 | 0.0 | 0.0 | 0.0 | 2.2 |  |
|  | Soto 2007[49] | C-S | | H | |  |  | Cells | MY+GP | HSIL | 45 | 55.6 | 31.1 | | 6.7 |  | 17.8 | | 2.2 | | 4.4 | | 15.6 |  |  | | |  | 4.4 |  | 4.4 | 8.9 | 0.0 |  | 11.1 | 2.2 |
| **Ecuador**  (1) 79 [1.0] | Paez 1996^[50]^ | C-S | | H | |  | 20-69 | Fixed bio. | MY/C | HSIL | 32 | 50.0 | 81.3 | | 6.3 |  |  | |  | |  | |  |  |  | | |  |  |  |  |  |  |  | 12.5 |  |
|  |  |  |  |  |  |  |  |  |  | ICC | 47 | 81.0 | 81.6 | | 5.3 |  |  | |  | |  | |  |  |  | | |  |  |  |  |  |  |  | 13.2 |  |
| **Honduras**  (1) 185 [2.3] | Ferrera 1999^[51]^ | C-C | | M | | 50.0 | 26-84 | Cells | MY/C | HSIL | 81 | 79.0 | 35.8 | | 7.4 | 3.7 | 8.6 | | 4.9 | | 7.4 | | 1.2 | 1.2 | 0.0 | | | 1.2 | 0.0 | 0.0 | 0.0 | 0.0 | 1.2 | 2.5 | 6.2 | 4.9 |
|  |  |  |  |  |  |  |  |  |  | ICC | 104 | 79.8 | 43.3 | | 10.6 | 5.8 | 3.8 | | 7.7 | | 6.7 | | 1.0 | 0.0 | 1.0 | | | 0.0 | 0.0 | 0.0 | 0.0 | 0.0 | 0.0 | 0.0 | 3.8 | 4.8 |
| **Jamaica**  (1) 66 [0.8] | Rattray 1996^[52]^ | C-S | | H | |  |  | Cells | MY/C | HSIL | 66 | 80.3 | 24.2 | | 4.5 | 13.6 | 9.1 | | 7.6 | |  | |  | 13.6 |  | | |  |  |  |  |  | 7.6 | 1.5 | 19.7 | 15.2 |
| **Mexico**  (16)  1432  [17.9] | Bermudez-Morales2008^[53]^ | C-S | | H | | 46.1 |  | Fixed Bio. | ISH | ICC | 50 | 84.0 | 66.0 | | 22.0 |  | 8.0 | |  | |  | |  |  |  | | |  |  |  |  |  |  |  |  | 2.0 |
|  | Berumen 2001^[54]^ | C-S | | H | |  | 26-89 | Fresh Bio. | MY/C | ICC | 181 |  | 50.8 | |  |  |  | |  | |  | |  |  |  | | |  |  |  |  |  |  |  |  |  |
|  | Carrillo 2004^[55]^ | C-S | | H | | 41.9 |  | Cells | MY+GP | HSIL | 22 | 91.7 | 58.3 | | 4.2 | 0.0 | 4.2 | | 8.3 | | 4.2 | | 0.0 | 0.0 | 0.0 | | |  | 4.2 |  | 4.2 | 0.0 | 0.0 | 0.0 | 4.2 |  |
|  |  |  |  |  |  | 47.1 |  |  |  | ICC | 44 | 95.5 | 50.0 | | 18.2 | 6.8 | 4.5 | | 0.0 | | 0.0 | | 2.3 | 0.0 | 2.3 | | |  | 2.3 |  | 0.0 | 2.3 | 0.0 | 0.0 | 4.5 |  |
|  | Fernandez 2007^[57]^ | C-S | | H | |  | 16-97 | Fixed Bio. | ISH | HSIL | 40 | 77.5 | 42.5 | | 2.5 | 0.0 | 7.5 | | 2.5 | | 0.0 | | 0.0 |  | 2.5 | | |  |  |  |  | 0.0 |  |  | 10.0 | 10.0 |
|  |  |  |  |  |  |  |  |  |  | ICC | 48 | 100.0 | 70.8 | | 12.5 | 0.0 | 6.3 | | 0.0 | | 2.1 | | 2.1 |  | 0.0 | | |  |  |  |  | 0.0 |  |  |  | 6.3 |
|  | Giuliano 2001^[56]^ | C-S | | H | | 33.1 | 15-79 | Cells | MY/C | HSIL | 22 | 59.1 | 27.3 | | 0.0 | 9.1 | 4.6 | | 0.0 | | 13.6 | | 0.0 | 4.6 | 0.0 | | | 0.0 | 9.1 | 0.0 | 0.0 | 4.6 | 0.0 | 0.0 | 0.0 |  |
|  | Gonzalez-Garay 1992^[58]^ | C-S | | H | |  |  | Cells | SB/DB/F | ICC | 37 |  | 27.0 | | 5.4 |  |  | |  | |  | |  |  |  | | |  |  |  |  |  |  |  |  | 4.2 |
|  | González-Losa 2004^[59]^ | C-S | | H | | 38.7 | 17-80 | Fresh Bio. | MY/C | HSIL | 67 |  | 26.7 | | 20.0 | 0.0 | 6.7 | | 6.7 | | 26.7 | | 0.0 | 6.7 | 0.0 | | | 0.0 | 6.7 |  | 0.0 | 0.0 | 0.0 | 0.0 | 0.0 |  |
|  |  |  |  |  |  |  |  |  |  | ICC | 15 | 92.5 | 25.4 | | 10.4 | 3.0 | 7.5 | | 14.9 | | 28.4 | | 0.0 | 0.0 | 6.0 | | | 7.5 | 4.5 |  | 4.5 | 6.0 | 1.5 | 4.5 |  | 22.4 |
|  | Hernandez-Avila 1997^[60]^ | C-C | | M | | 44.9 | 25-75 | Cells | E6/E7/O | HSIL | 60 |  | 48.8 | | 11.4 |  |  | |  | |  | |  |  |  | | |  |  |  |  |  |  |  |  |  |
|  |  |  |  |  |  |  |  |  |  | ICC | 88 |  | 48.3 | | 0.0 |  |  | |  | |  | |  |  |  | | |  |  |  |  |  |  |  |  |  |
|  | Illades-Aguiar 2009^[61]^ | C-C | | M | |  |  | Cell+Fresh | MY/C | ICC | 133 | 66.9 | 9.0 | | 4.5 | 7.5 | 0.8 | | 0.8 | | 1.5 | |  | 0.0 | 0.8 | | |  |  |  |  | 0.8 | 0.8 |  |  | 66.9 |
|  | Lazcano-Ponce 2009^[62]^ | RCT | | M | | 20.7 | 18-23 | Fixed Bio. | PCR | HSIL | 6 |  | 83.3 | |  |  |  | |  | |  | |  |  |  | | |  |  |  |  |  | 16.7 |  |  |  |
|  | Lizano 2006^[63]^ | C-S | | H | |  |  | Fresh Bio. | GP5/6 | HSIL | 47 | 57.4 | 48.9 | | 8.5 |  |  | |  | |  | |  |  |  | | |  |  |  |  |  |  |  |  |  |
|  |  |  |  |  |  |  |  |  |  | ICC | 135 | 65.9 | 50.3 | | 15.5 |  |  | |  | |  | |  |  |  | | |  |  |  |  |  |  |  |  |  |
|  | Lopez-Revilla 2008^[64]^ | CS | | H | |  | 16-78 | Cells | MY/C | HSIL | 64 |  | 82.8 | | 7.8 |  | 18.8 | | 3.1 | |  | | 9.4 | 9.4 |  | | |  |  |  |  |  |  |  |  | 29.7 |
|  |  |  |  |  |  |  |  |  |  | ICC | 39 |  | 74.4 | | 5.1 |  | 35.9 | | 5.1 | |  | | 5.1 | 10.3 |  | | |  |  |  |  |  |  |  |  |  |
|  | Matthews-Greer 2004^[65]^ | C-S | | H | |  |  | Fixed Bio. | MY/C | ICC | 14 | 100.0 | 71.4 | | 21.4 | 0.0 | 7.1 | | 7.1 | | 0.0 | | 0.0 | 0.0 | 0.0 | | | 0.0 | 0.0 | 7.1 | 7.1 | 0.0 | 0.0 | 0.0 | 0.0 | 21.4 |
|  | Meyer 1998^[66]^ | C-S | | H | |  |  | Fixed Bio. | ISH | ICC | 60 | 96.7 | 43.3 | | 16.7 | 3.3 | 23.3 | | 6.7 | | 0.0 | | 1.7 | 1.7 | 0.0 | | | 1.7 | 0.0 | 0.0 | 1.7 | 0.0 | 3.3 | 0.0 |  |  |
|  | Piña-Sanchez 2006^[67]^ | C-S | | H | | 40.9 | 19–96 | Cells | MY/C | HSIL | 59 | 88.1 | 42.2 | | 6.7 | 1.7 | 3.1 | | 1.7 | | 18.6 | | 3.4 | 0.0 | 0.0 | | | 3.4 |  |  | 1.7 | 1.7 | 10.2 | 0.0 | 10.2 |  |
|  |  |  |  |  |  |  |  |  |  | ICC | 108 | 91.6 | 72.2 | | 4.6 | 0.9 | 1.9 | | 0.9 | | 1.9 | | 0.0 | 0.9 | 1.9 | | | 0.0 |  |  | 0.9 | 0.0 | 6.5 | 0.0 | 11.1 |  |
|  | Torroella-Kouri 1998^[68]^ | C-S | | H | | 47.3 | 15-85 | Cell+Fresh | MY/C | HSIL | 24 | 83.3 | 58.3 | | 12.5 | 0.0 | 0.0 | | 8.3 | | 12.5 | | 0.0 |  | 0.0 | | | 4.2 | 4.2 | 0.0 | 0.0 | 0.0 |  |  | 12.5 |  |
|  |  |  |  |  |  |  |  |  |  | ICC | 69 | 87.0 | 42.0 | | 14.5 | 10.1 | 2.9 | | 0.0 | | 4.3 | | 2.9 |  | 4.3 | | | 1.4 | 0.0 | 1.4 | 4.3 | 1.4 |  |  | 4.3 |  |
| **Nicaragua**  (3)  311  [3.9] | Aerssens 2008^[69]^ | PFU | | M | | 34.3 |  | Cell+Fresh | SPF | HSIL | 67 | 100.0 | 31.3 | |  |  | 17.9 | |  | | 16.4 | | 14.9 |  |  | | |  |  |  |  |  |  |  | 7.5 | 29.9 |
|  | Aerssens 2009^[70]^ | PFU | | M | | 34.8 | 20-60 | Cell+Fresh | SPF | ICC | 117 |  | 28.2 | |  |  | 16.2 | |  | | 9.4 | | 15.4 |  |  | | |  | 10.3 |  |  |  |  |  |  | 33.3 |
|  | Hindryckx 2006^[71]^ | C-S | | H | | 36.7 | 17-69 | Cells | E6/E7/O | HSIL | 108 |  | 26.9 | | 6.5 | 3.7 | 12.0 | | 5.6 | | 14.8 | | 11.1 | 2.8 | 2.8 | | | 1.9 | 6.5 | 4.6 | 4.6 | 0.9 |  |  | 1.9 |  |
|  |  |  |  |  |  |  |  |  |  | ICC | 19 |  | 52.6 | | 5.3 | 5.3 | 10.5 | | 5.3 | | 5.3 | | 5.3 | 0.0 | 0.0 | | | 10.5 | 5.3 | 5.3 | 0.0 | 0.0 |  |  | 5.3 |  |
| **Panama**  (2)255[3.2] | Acs 1989^[72]^ | C-S | | H | |  | 18+ | Fixed bio. | SB/DB/F | ICC | 182 | 62.0 | 37.0 | |  |  |  | |  | |  | |  |  |  | | |  |  |  |  |  |  |  |  |  |
|  | Bosch (2004) 1995^[5]*^ | C-S | | M | |  |  | Fixed bio. | ISH | ICC | 73 | 100.0 | 47.9 | | 15.1 | 9.6 | 4.1 | | 1.4 | | 4.1 | | 4.1 | 2.7 | 6.8 | | | 0.0 | 2.7 | 0.0 | 0.0 | 0.0 | 1.4 | 1.4 | 2.7 |  |
| **Paraguay**  (2) 228  [2.9] | Mendoza 2010^[74]^ | C-S | H | |  | | 16-77 | Fresh Bio. | SB/DB/F | HSIL | 74 |  | | 41.9 | 1.4 | 2.7 | | 6.8 | | 12.2 | | 5.4 | 9.5 | 1.4 | | 2.7 | 4.1 | | 6.8 |  |  | 2.7 | 2.7 | 2.7 |  |  |
|  |  |  |  |  |  |  |  |  |  | ICC | 41 |  | | 75.6 | 2.4 | 4.9 | | 12.2 | | 22.0 | | 9.8 | 17.1 | 2.4 | | 4.9 | 7.3 | | 12.2 |  |  | 4.9 | 4.9 |  |  |  |
|  | Rolon 2000^[73]^ | C-C | H | | 49.0 | | 18-85 | Fresh bio. | GP5/6 | ICC | 113 | 96.5 | | 47.8 | 10.1 | 3.5 | | 3.6 | | 4.4 | | 2.7 | 0.9 | 1.8 | | 1.8 |  | |  |  | 0.9 |  |  |  | 19.5 | 18.6 |
| **Peru**  (2) 231  [2.9] | Lorincz 1986^[75]^ | C-S | H | |  | |  | Fixed Bio. | SB/DB/F | HSIL | 10 |  | |  |  |  | | 20.0 | |  | |  |  |  | |  |  | |  |  |  |  |  |  |  |  |
|  |  |  |  |  |  |  |  |  |  | ICC | 23 |  | |  |  |  | | 8.7 | |  | |  |  |  | |  |  | |  |  |  |  |  |  |  |  |
|  | Santos 2001^[76]^ | C-S | H | |  | |  | Fresh Bio. | GP5/6 | ICC | 198 |  | | 55.6 | 12.6 | 4.5 | | 10.1 | | 1.0 | | 2.0 | 6.1 | 3.5 | | 0.5 | 1.0 | | 1.0 | 0.0 | 2.0 | 1.0 | 0.0 | 0.0 |  | 12.1 |
| **Surinam**  (2)246[3.1] | De Boer 2004^[77]^ | C-S | H | |  | |  | Fixed Bio. | MY/C | ICC | 116 | 88.8 | | 35.3 | 17.2 |  | |  | |  | |  |  |  | |  |  | |  |  |  |  |  |  |  | 17.2 |
|  | Krul 1999^[78]^ | C-S | H | | 47.0 | | 23-98 | Fixed Bio. | MY+GP | ICC | 130 | 82.0 | | 49.0 | 15.0 |  | |  | |  | |  |  |  | |  |  | |  |  |  |  |  |  |  |  |
| **By Country (S):** Number of studies0substudies **N**: Number of samples/patients**, [%]** Percentage out of the total  **C-S**: Cross-sectional; **CC**: Case-control; **PFU**: prospective follow-up study; **RCT**: Randomized Controlled Trial; **H**: High; **M**: Moderate; **Cells**: Exfoliated cells; **Fixed Bio.**: Fixed Biopsies; **Fresh Bio.**: Fresh Biopsies; **SB/DB/F** Southern blot, Dot blot, FISH; **ISH**: In Situ Hybridization; **MY/C**: MY09/11 or Consensus primers; **E6, E7**: E6, E7, others; **HSIL**: Highgrade Squamous Intraepithelial Lesions; **ICC**: Invasive Cervical Cancer  * Bosch (2004) 1995^[5]^ is presented as 7 substudies by country. | | | | | | | | | | | | | | | | | | | | | | | | | | | | | | | | | | | |  |

**References**

1. Abba MC, Gomez MA, Golijow CD (2003) [Human papillomavirus genotype distribution in cervical infections among woman in La Plata, Argentina]. Rev Argent Microbiol 35: 74-79.

2. Alonio LV, Dalbert D, Mural J, Bartt O, Bazan G, et al. (1990) Different papillomaviruses in uterine cervical lesions: Detection and location by 'in situ' hydridization with biotinylated probes. Cervix & the Lower Female Genital Tract 8: 339-348.

3. Alonio LV, Dalbert D, Picconi MA, Cervantes Vazquez G, Garcia Carranca A, et al. (2000) [Ha-ras and p53 gene mutations scanned by PCR-SSCP in premalignant and malignant lesions of the uterine cervix associated with human papillomavirus]. Medicina (B Aires) 60: 895-901.

4. Alonio LV, Picconi MA, Dalbert D, Mural J, Bartt O, et al. (2003) Ha-ras oncogene mutation associated to progression of papillomavirus induced lesions of uterine cervix. J Clin Virol 27: 263-269.

5. Bosch FX, Manos MM, Munoz N, Sherman M, Jansen AM, et al. (1995) Prevalence of human papillomavirus in cervical cancer: A worldwide perspective. Journal of the National Cancer Institute 87: 796-802.

6. Chouhy D, Gil LB, Nocito AL, Wojdyla D, Ornella L, et al. (2006) Development and evaluation of a colorimetric PCR system for the detection and typing of human papillomaviruses. Int J Mol Med 18: 995-1003.

7. Deluca GD, Lucero RH, Martin de Civetta MT, Vicente L, de Gorodner OL, et al. Genotipificación del Virus Papiloma Humano (HPV) por PCR-RFLP en alteraciones cervicales; 2002.

8. Deluca GD, Marin HM, Schelover E, Chamorro EM, Vicente L, et al. (2006) Chlamydia trachomatis and papillomavirus infection in women with cytohistological abnormalities in uterine cervix. [Spanish]. Medicina 66: 303-306.

9. Golijow CD, Abba MC, Mouron SA, Laguens RM, Dulout FN, et al. (2005) Chlamydia trachomatis and Human papillomavirus infections in cervical disease in Argentine women. Gynecol Oncol 96: 181-186.

10. Jantus Lewintre E MdCM (1998) Cancer de cuello uterino en Corrientes (Argentina): Tipificación de virus del papiloma humano (HPV) en lesiones cervicales por PCR-Hibridación. Nuevas tendencias en Oncología 7: 134-139.

11. Perez LO, Barbisan G, Abba MC, Laguens RM, Dulout FN, et al. (2006) Herpes simplex virus and human papillomavirus infection in cervical disease in Argentine women. Int J Gynecol Pathol 25: 42-47.

12. Picconi MA, Alonio LV, Garcia Carranca A, Lizano M, Cervantes Vazquez G, et al. (2000) [Molecular variants of human papillomavirus (HPV) types 16 and 18 in adenocarcinomas of the cervix]. Medicina (B Aires) 60: 889-894.

13. Picconi MA, Gronda J, Alonio LV, Villa LL, Sichero L, et al. (2002) Virus papiloma humano en mujeres quechuas jujeñas con alta frecuencia de cancer de cuello uterino: tipos virales y variantes de HPV 16. Medicina (BAires) 62: 209-220.

14. Tatti S, Fleider L, Tinnirello M, Chabelski C, Vighi S, et al. (2007) AVANCES EN LA PREVENCION DEL CANCER CERVICAL: “del PAPANICOLAOU a la VACUNA PROFILACTICA para el PAPILOMAVIRUS HUMANO (HPV)”. Buenos Aires.

15. Tonon SA, Picconi MA, Zinovich JB, Liotta DJ, Bos PD, et al. (1999) Human papillomavirus cervical infection and associated risk factors in a region of Argentina with a high incidence of cervical carcinoma. Infect Dis Obstet Gynecol 7: 237-243.

16. Prussia PR, Schegget J, Smits HL (1993) Detection of oncogenic HPV DNA by a consensus polymerase chain reaction method in genital carcinomas in twenty women in Barbados. West Indian med j 42: 144-146.

17. Cathro HP, Loya T, Dominguez F, Howe SL, Howell R, et al. (2009) Human papillomavirus profile of women in Belize City, Belize: correlation with cervical cytopathologic findings. Human Pathology 40: 942-949.

18. Armbruster-Moraes E, Ioshimoto LM, Leao E, Zugaib M (2000) Prevalence of 'high risk' human papillomavirus in the lower genital tract of Brazilian gravidas. Int J Gynaecol Obstet 69: 223-227.

19. Baldez da Silva MF, Chagas BS, Guimaraes V, Katz LM, Felix PM, et al. (2009) HPV31 and HPV33 incidence in cervical samples from women in Recife, Brazil. Genet Mol Res 8: 1437-1443.

20. Bagarelli LB, Oliani AH (2004) Tipagem e estado físico de papilomavírus humano por hibridização in situ em lesões intra-epiteliais do colo uterino. Rev bras ginecol obstet 26: 59-64.

21. Cambruzzi E, Zettler CG, Alexandre CO (2005) Expression of Ki-67 and squamous intraepithelial lesions are related with HPV in endocervical adenocarcinoma. Pathol Oncol Res 11: 114-120.

22. Cavalcanti SM, Frugulhetti IC, Passos MR, Fonseca ME, Oliveira LH (1994) Prevalence of human papillomavirus DNA in female cervical lesions from Rio de Janeiro, Brazil. Mem Inst Oswaldo Cruz 89: 575-580.

23. Cavalcanti SM, Deus FC, Zardo LG, Frugulhetti IC, Oliveira LH (1996) Human papillomavirus infection and cervical cancer in Brazil: a retrospective study. Mem Inst Oswaldo Cruz 91: 433-440.

24. Eluf-Neto J, Booth M, Munoz N, Bosch FX, Meijer CJ, et al. (1994) Human papillomavirus and invasive cervical cancer in Brazil. Br J Cancer 69: 114-119.

25. Fernandes JV, de Vasconcellos Meissner R, de Carvalho MGF, de Medeiros Fernandes TAA, de Azevedo PRM, et al. (2009) Prevalence of HPV infection by cervical cytologic status in Brazil. International Journal of Gynecology and Obstetrics 105: 21-24.

26. Franco E, Villa L, Rohan T, Ferenczy A, Petzl-Erler M, et al. (1999) Design and methods of the Ludwig-McGill longitudinal study of the natural history of human papillomavirus infection and cervical neoplasia in Brazil. Ludwig-McGill Study Group. Rev Panam Salud Publica 6: 223-233.

27. Freitas TP, Carmo BBd, Paula FDF, Rodrigues LF, Fernandes AP, et al. (2007) Molecular detection of HPV 16 and 18 in cervical samples of patients from Belo Horizonte, Minas Gerais, Brazil. Rev Inst Med Trop SAo Paulo 49: 297-301.

28. Lorenzato F, Ho L, Terry G, Singer A, Santos LC, et al. (2000) The use of human papillomavirus typing in detection of cervical neoplasia in Recife (Brazil). International Journal of Gynecological Cancer 10: 143-150.

29. Maciag PC, Schlecht NF, Souza PS, Franco EL, Villa LL, et al. (2000) Major histocompatibility complex class II polymorphisms and risk of cervical cancer and human papillomavirus infection in Brazilian women. Cancer Epidemiol Biomarkers Prev 9: 1183-1191.

30. Noronha V, Mello W, Villa L, Brito A, Macêdo R, et al. (1999) Papilomavírus humano associado a lesöes de cérvice uterina. Rev Soc Bras Med Trop 32: 235-240.

31. Oliveira LdHdS, Rodrigues EdVM, Lopes APTAdS, Fernandez AdP, Cavalcanti SMB (2003) HPV 16 detection in cervical lesions, physical state of viral DNA and changes in p53 gene. SAo Paulo med j 121: 67-71.

32. Oliveira LHS, Rosa MLG, Pereira CRN, Vasconcelos GALBM, Silva RA, et al. (2006) Human papillomavirus status and cervical abnormalities in women from public and private health care in Rio de Janeiro State, Brazil. Rev Inst Med Trop São Paulo 48: 279-285.

33. Pinheiro NA, Villa LL (2001) Low frequency of p53 mutations in cervical carcinomas among Brazilian women. Braz j med biol res = Rev bras pesqui m‚d biol 34: 727-733.

34. Rabelo-Santos SH, Zeferino L, Villa LL, Sobrinho JP, Amaral RG, et al. (2003) Human papillomavirus prevalence among women with cervical intraepithelial neoplasia III and invasive cervical cancer from Goiania, Brazil. Mem Inst Oswaldo Cruz 98: 181-184.

35. Rabelo-Santos SH, Derchain SF, Villa LL, Costa MC, Sarian LO, et al. (2009) Human papillomavirus-specific genotypes in cervical lesions of women referred for smears with atypical glandular cells or adenocarcinoma in situ. Int J Gynecol Pathol 28: 272-278.

36. Souen J, Ramos LO, Motta E, Eluf Neto J (1995) Prevalência de híbridos do HPV entre portadoras de carcinoma do colo do útero. Rev bras ginecol obstet 17: 509-512.

37. Terra AP, Murta EF, Maluf PJ, Caballero OL, Brait M, et al. (2007) Aberrant promoter methylation can be useful as a marker of recurrent disease in patients with cervical intraepithelial neoplasia grade III. Tumori 93: 572-579.

38. Aedo A S, Melo A A, García P, Guzmán G P, Capurro V I, et al. (2007) Detección y tipificación de virus papiloma humano en lesiones preneoplásicas del cuello uterino mediante PCR-RFLP. Rev méd Chile 135: 167-173.

39. Melo A, Montenegro S, Hooper T, Capurro I, Roa JC, et al. (2003) [Human papilloma virus (HPV) typing in preneoplastic and neoplastic lesions of the uterine cervix in the IX region-Chile]. Rev Med Chil 131: 1382-1390.

40. Roa S JC, Martínez S R, Montenegro S, Roa E I, Capurro V I, et al. (2007) Inestabilidad microsatelital en lesiones preneoplásicas y neoplásicas del cuello uterino: Correlación con el genotipo del virus papiloma humano. Rev méd Chile 135: 37-44.

41. Roa JC, Garcia P, Gomez J, Fernandez W, Gaete F, et al. (2009) HPV genotyping from invasive cervical cancer in Chile. Int J Gynaecol Obstet 105: 150-153.

42. Bosch FX, Munoz N, de Sanjose S, Navarro C, Moreo P, et al. (1993) Human papillomavirus and cervical intraepithelial neoplasia grade III/carcinoma in situ: a case-control study in Spain and Colombia. Cancer Epidemiol Biomarkers Prev 2: 415-422.

43. Molano M, Moreno Acosta P, Bravo MM (2007) Types and variants of human papillomavirus in patients with cervical cancer submitted to radiotherapy. Biosalud: 45-57.

44. Munoz N, Bosch FX, de Sanjose S, Tafur L, Izarzugaza I, et al. (1992) The causal link between human papillomavirus and invasive cervical cancer: a population-based case-control study in Colombia and Spain. Int J Cancer 52: 743-749.

45. Muñoz N, Hernandez-Suarez G, Mendez F, Molano M, Posso H, et al. (2009) Persistence of HPV infection and risk of high-grade cervical intraepithelial neoplasia in a cohort of Colombian women. British Journal of Cancer 100: 1184-1190.

46. Murillo R, Molano M, Martinez G, Mejia JC, Gamboa O (2009) HPV prevalence in Colombian women with cervical cancer: implications for vaccination in a developing country. Infect Dis Obstet Gynecol 2009: 653598.

47. Sierra-Torres CH, Arboleda-Moreno YY, Orejuela-Aristizabal L (2006) Exposure to wood smoke, HPV infection, and genetic susceptibility for cervical neoplasia among women in Colombia. Environ Mol Mutagen 47: 553-561.

48. Schiffman M, Herrero R, Desalle R, Hildesheim A, Wacholder S, et al. (2005) The carcinogenicity of human papillomavirus types reflects viral evolution. Virology 337: 76-84.

49. Soto Y, Mune M, Morales E, Goicolea A, Mora J, et al. (2007) Human papillomavirus infections in Cuban women with cervical intraepithelial neoplasia. Sexually Transmitted Diseases 34: 974-976.

50. Paez C, Konno R, Yaegashi N, Matsunaga G, Araujo I, et al. (1996) Prevalence of HPV DNA in cervical lesions in patients from Ecuador and Japan. Tohoku J Exp Med 180: 261-272.

51. Ferrera A, Velema JP, Figueroa M, Bulnes R, Toro LA, et al. (1999) Human papillomavirus infection, cervical dysplasia and invasive cervical cancer in Honduras: a case-control study. Int J Cancer 82: 799-803.

52. Rattray C, Strickler HD, Escoffery C, Cranston B, Brown C, et al. (1996) Type-specific prevalence of human papillomavirus DNA among Jamaican colposcopy patients. J Infect Dis 173: 718-721.

53. Bermudez-Morales VH, Gutierrez LX, Alcocer-Gonzalez JM, Burguete A, Madrid-Marina V (2008) Correlation between IL-10 gene expression and HPV infection in cervical cancer: a mechanism for immune response escape. Cancer Invest 26: 1037-1043.

54. Berumen J, Ordonez RM, Lazcano E, Salmeron J, Galvan SC, et al. (2001) Asian-American variants of human papillomavirus 16 and risk for cervical cancer: a case-control study. J Natl Cancer Inst 93: 1325-1330.

55. Carrillo A, Mohar A, Meneses A, Frias-Mendivil M, Solorza G, et al. (2004) Usefulness of combining universal oligonucleotides in detecting human papillomavirus in cervical cancer and premalignant lesions. [Spanish]. Salud Publica de Mexico 46: 7-15.

56. Giuliano AR, Papenfuss M, Abrahamsen M, Denman C, de Zapien JG, et al. (2001) Human papillomavirus infection at the United States-Mexico border: implications for cervical cancer prevention and control. Cancer Epidemiol Biomarkers Prev 10: 1129-1136.

57. Fernandez-Tilapa G, Illades-Aguiar B, Martinez-Carrillo DN, del Carmen Alarcon-Romero L, Vences-Velazquez A, et al. (2007) Prevalence of human papillomavirus types among Mexican women with intraepithelial lesions and cervical cancer: Detection with MY09/MY011 and GP5+/GP6+ primer systems. American Journal of Infectious Diseases 3: 62-67.

58. Gonzalez-Garay ML, Barrera-Saldana HA, Aviles LB, Alvarez-Salas LM, Gariglio P (1992) Prevalence in two mexican cities of human papillomavirus DNA sequences in cervical cancer. Rev Invest Clin 44: 491-499.

59. Gonzalez-Losa MDR, Rosado-Lopez I, Valdez-Gonzalez N, Puerto-Solis M (2004) High prevalence of human papillomavirus type 58 in Mexican colposcopy patients. Journal of Clinical Virology 29: 202-205.

60. Hernandez-Avila M, Lazcano-Ponce EC, Berumen-Campos J, Cruz-Valdez A, Alonso de Ruiz PP, et al. (1997) Human papilloma virus 16-18 infection and cervical cancer in Mexico: a case-control study. Arch Med Res 28: 265-271.

61. Illades-Aguiar B, Cortes-Malagon EM, Antonio-Vejar V, Zamudio-Lopez N, Alarcon-Romero LdC, et al. (2009) Cervical carcinoma in Southern Mexico: Human papillomavirus and cofactors. Cancer Detection and Prevention 32: 300-307.

62. Lazcano-Ponce E, Perez G, Cruz-Valdez A, Zamilpa L, Aranda-Flores C, et al. (2009) Impact of a Quadrivalent HPV6/11/16/18 Vaccine in Mexican Women: Public Health Implications for the Region. Archives of Medical Research 40: 514-524.

63. Lizano M, De la Cruz-Hernandez E, Carrillo-Garcia A, Garcia-Carranca A, Ponce de Leon-Rosales S, et al. (2006) Distribution of HPV16 and 18 intratypic variants in normal cytology, intraepithelial lesions, and cervical cancer in a Mexican population. Gynecologic Oncology 102: 230-235.

64. Lopez-Revilla R, Martinez-Contreras LA, Sanchez-Garza M (2008) Prevalence of high-risk human papillomavirus types in Mexican women with cervical intraepithelial neoplasia and invasive carcinoma. Infectious Agents and Cancer 3.

65. Matthews-Greer J, Dominguez-Malagon H, Herrera GA, Unger J, Chanona-Vilchis J, et al. (2004) Human Papillomavirus Typing of Rare Cervical Carcinomas. Archives of Pathology & Laboratory Medicine 128: 553-556.

66. Meyer T, Arndt R, Christophers E, Beckmann ER, Schroder S, et al. (1998) Association of rare human papillomavirus types with genital premalignant and malignant lesions. J Infect Dis 178: 252-255.

67. Pinña-Sanchez P, Hernandez-Hernandez DM, Lopez-Romero R, Vazquez-Ortiz G, Perez-Plasencia C, et al. (2006) Human papillomavirus-specific viral types are common in Mexican women affected by cervical lesions. International Journal of Gynecological Cancer 16: 1041-1047.

68. Torroella-Kouri M, Morsberger S, Carrillo A, Mohar A, Meneses A, et al. (1998) HPV prevalence among Mexican women with neoplastic and normal cervixes. Gynecol Oncol 70: 115-120.

69. Aerssens A, Claeys P, Garcia A, Sturtewagen Y, Velasquez R, et al. (2008) Natural history and clearance of HPV after treatment of precancerous cervical lesions. Histopathology 52: 381-386.

70. Aerssens A, Claeys P, Beerens E, Garcia A, Weyers S, et al. (2009) Prediction of recurrent disease by cytology and HPV testing after treatment of cervical intraepithelial neoplasia. Cytopathology 20: 27-35.

71. Hindryckx P, Garcia A, Claeys P, Gonzalez C, Velasquez R, et al. (2006) Prevalence of high risk human papillomavirus types among Nicaraguan women with histological proved pre-neoplastic and neoplastic lesions of the cervix. Sex Transm Infect 82: 334-336.

72. Acs J, Hildesheim A, Reeves WC, Brenes M, Brinton L, et al. (1989) Regional distribution of human papillomavirus DNA and other risk factors for invasive cervical cancer in Panama. Cancer Res 49: 5725-5729.

73. Rolon PA, Smith JS, Munoz N, Klug SJ, Herrero R, et al. (2000) Human papillomavirus infection and invasive cervical cancer in Paraguay. Int J Cancer 85: 486-491.

74. Mendoza LP, Arbiza J, Paez M, Kasamatsu E, Castro A, et al. (2011) Distribution of human papillomavirus genotypes in Paraguayan women according to the severity of the cervical lesion. J Med Virol 83: 1351-1357.

75. Lorincz AT, Lancaster WD, Temple GF (1986) Cloning and characterization of the DNA of a new human papillomavirus from a woman with dysplasia of the uterine cervix. Journal of Virology 58: 225-229.

76. Santos C, Munoz N, Klug S, Almonte M, Guerrero I, et al. (2001) HPV types and cofactors causing cervical cancer in Peru. Br J Cancer 85: 966-971.

77. de Boer MA, Peters LA, Aziz MF, Siregar B, Cornain S, et al. (2004) Human papillomavirus type 16 E6, E7, and L1 variants in cervical cancer in Indonesia, Suriname, and The Netherlands. Gynecol Oncol 94: 488-494.

78. Krul EJT, Van De Vijver MJ, Schuuring E, Van Kanten RW, Peters AAW, et al. (1999) Human papillomavirus in malignant cervical lesions in Surinam a high- risk country, compared to the Netherlands, a low-risk country. International Journal of Gynecological Cancer 9: 206-211.
