# Supplementary material for: Type-Specific HPV Prevalence in Cervical Cancer and High-Grade Lesions in Latin America and the Caribbean: Systematic Review and Meta-Analysis
Source: PLoS One. 2011 Oct 4;6(10):e25493. doi: 10.1371/journal.pone.0025493 (PMC3186785; doi:10.1371/journal.pone.0025493)
Supplement: Diagram S1 — PRISMA study flow diagram for reporting systematic reviews and meta-analyse. (DOC) [file pone.0025493.s005.doc]

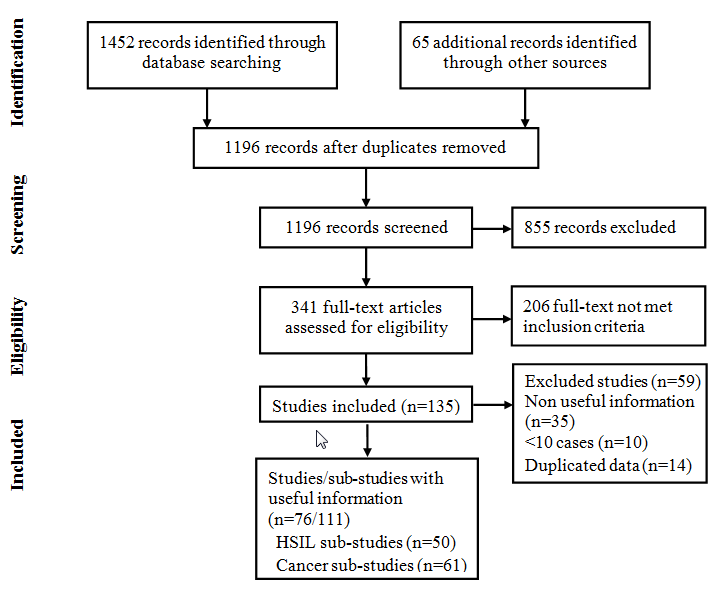


Potentially relevant studies (n=1452)

Duplicated studies (n=256)

Potentially relevant single

studies (n=1196)

Excluded studies because not met screening criteria (n=855)

Studies’ full text retrieved for detailed evaluation (n=341)

Studies included (n=135)

**Excluded studies (n=56)**

Non useful information (n=32)

<10 cases (n=10)

Duplicated data (n=14)

**Studies/sub-studies with useful information (n=79/114)**

HSIL sub-studies (n=52)

Cancer sub-studies (n=62)

**Figure 1** **– Study flow diagram**
